# Poly(acrylic acid-co-acrylamide)/Polyacrylamide pIPNs/Magnetite Composite Hydrogels: Synthesis and Characterization

**DOI:** 10.3390/gels9050365

**Published:** 2023-04-26

**Authors:** Marin Simeonov, Anton Atanasov Apostolov, Milena Georgieva, Dimitar Tzankov, Elena Vassileva

**Affiliations:** 1 Laboratory on Structure and Properties of Polymers, Faculty of Chemistry and Pharmacy, University of Sofia “St. Kliment Ohridski”, 1, James Bourchier blvd., 1164 Sofia, Bulgaria; 2 Faculty of Physics, University of Sofia “St. Kliment Ohridski”, 5, James Bourchier blvd., 1164 Sofia, Bulgaria

**Keywords:** magnetite, polyacrylamide, poly(acrylic acid), interpenetrating polymer networks, hydrogels, polymer nanocomposites

## Abstract

Novel composite hydrogels based on poly(acrylic acid-co-acrylamide)/polyacrylamide pseudo-interpenetrating polymer networks (pIPNs) and magnetite were prepared via in situ precipitation of Fe^3+^/Fe^2+^ ions within the hydrogel structure. The magnetite formation was confirmed by X-ray diffraction, and the size of the magnetite crystallites was shown to depend on the hydrogel composition: the crystallinity of the magnetite particles increased in line with PAAM content within the composition of the pIPNs. The Fourier transform infrared spectroscopy revealed an interaction between the hydrogel matrix, via the carboxylic groups of polyacrylic acid, and Fe ions, which strongly influenced the formation of the magnetite articles. The composites’ thermal properties, examined using differential scanning calorimetry (DSC), show an increase in the glass transition temperature of the obtained composites, which depends on the PAA/PAAM copolymer ratio in the pIPNs’ composition. Moreover, the composite hydrogels exhibit pH and ionic strength responsiveness as well as superparamagnetic properties. The study revealed the potential of pIPNs as matrices for controlled inorganic particle deposition as a viable method for the production of polymer nanocomposites.

## 1. Introduction

Hydrogels are three-dimensional networks built of hydrophilic polymers which can absorb and retain large amounts of water. Due to their high water content and soft nature, they are very similar to living tissues and thus possess very good biocompatibility. Therefore, they are extensively studied for biomedical applications such as drug delivery systems [1], biosensors [2], tissue engineering [3], etc. The combination of different polymers has the potential to ensure the development of novel polymeric materials with improved functionality. One of the possible ways to combine polymers of different natures and with different properties in one material is the formation of interpenetrating polymer networks (IPNs). IPNs comprise two or more networks that are at least partially interlaced on a molecular scale but not covalently bonded to each other and cannot be separated unless chemical bonds are broken [4]. When the IPN comprises a polymer network formed in the presence of a linear polymer, the so-called pIPNs are obtained [5].

Magnetite (Fe_3_O_4_) particles are used for various applications such as drug delivery, and bioseparation, and as contrast agents [6]. The major problem which arises when processing magnetic particles is their colloidal instability and tendency to aggregate due to dipole–dipole interactions [7]. To overcome their aggregation, the magnetite particles are either coated with polymers [8,9] or surface functionalized, e.g., with acids [10] or bioactive compounds [11]. Another possible approach to address this challenge is the in situ formation of magnetic particles within a polymer matrix, e.g., in a hydrogel [9,12,13], the latter being used as a template which also provides the possibility of controlling the particles’ size as well as the size distribution. This approach is inspired by nature as it is known that some bacteria are able to synthesize magnetic particles intracellularly using the in situ approach [14].

Poly(acrylic acid) (PAA) and polyacrylamide (PAAM) are among the polymers used for coating magnetite particles [8] due to their functionality. Both PAA and PAAM are known to interact with iron ions [9,10], providing centers for nucleation and crystal growth via their functional groups. When PAA and PAAM are combined in one material, e.g., as in copolymers or IPNs, hydrogen bonds are formed between their pendant carboxylic and amide groups, respectively. Thus, the PAA/PAAM-based materials are known to exhibit upper critical solution temperature (UCST) behavior due to these hydrogen bonds. These materials can respond simultaneously to changes in environmental pH (due to the COOH groups in the PAA) and temperature (due to the hydrogen bonds between both polymers), which defines them as smart materials.

Linear PAA was applied as a coating to stabilize magnetite nanoparticles [8,15] and prevent their agglomeration. Sanchez et al. study the effect of molecular weight and PAA content on the magnetic and structural properties of iron oxide nanoparticles. The results show that the increase in PAA content results in smaller sizes and narrower size distributions of the synthesized iron oxide particles. Magnetization analysis shows that the PAA-coated particles are superparamagnetic [8]. Chełminiak et. al. synthesized PAA-stabilized magnetite nanoparticles through photopolymerization. The synthesized particles were characterized by their pH sensitivity and superparamagnetic properties, which allow their fast separation from the media [15]. Several studies show that the PAA-coated magnetic particles can be useful materials for biomedical applications. The immobilization of PAA potentiates the anticoagulant activity and increases the thrombin time [16] as well as providing good biocompatibility [17] depending on the magnetic particles’ sizes. Large particles with high PAA content cause a reduction in proliferation of cell cultures, while smaller particles are not harmful to cell proliferation. PAAM hydrogels were explored as matrices for in situ formation of magnetite [12,13]. It was demonstrated that the water uptake and drug release rate of these hydrogels can be controlled by applying an external magnetic field. This is due to the alignment of magnetic moments of individual magnetite nanoparticles, which leads to the hydrogel swelling at a faster rate [12]. A current study demonstrates the coating of magnetite particles with a PAA/PAAM copolymer using gamma radiation. The pH- and temperature-responsiveness of the resulting hydrogels appear to depend on the hydrogels’ composition, i.e., the PAA/PAAM ratio. The resulting materials appear to be superparamagnetic. These hydrogels were successfully tested as drug delivery systems for doxorubicin [18].

To the best of our knowledge, however, no study to date has been dedicated to the role of IPN hydrogels combining both polymers in the in situ formation of magnetic particles. Thus, the aim of the study was to evaluate the role of P(AA-co-AAM)/PAAM pIPNs for the in situ formation of magnetite particles and therefore obtain novel P(AA-co-AAM)/PAAM pIPNs/magnetite composite hydrogels. We assume that the pIPNs’ composition and structure allow the control of the overall polymer network density and functionality and hence they are expected to influence the size and size distribution of the magnetite particles deposited in situ when compared to the single PAA and PAAM networks.

## 2. Results and Discussion

### 2.1. Swelling Behavior

#### Equilibrium Swelling Ratio (ESR)

**Figure 1:**
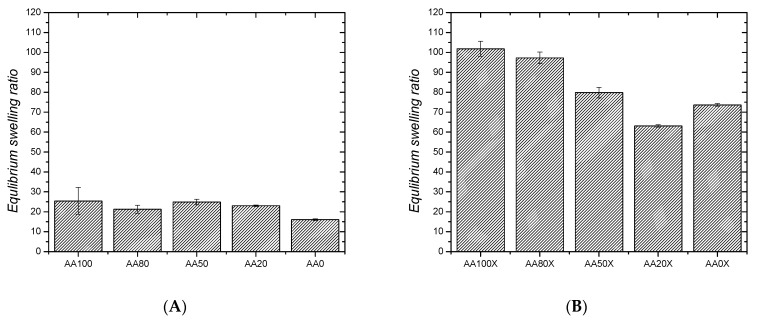
Equilibrium swelling ratio of: (**A**) neat pIPNs hydrogels and (**B**) pIPNs/magnetite composites.

The neat pIPNs hydrogels increase their initial weight ~20 times upon swelling in water (ESR~20–24) but no clear dependence of their ESR on the PAA/PAAM ratio is seen (Figure 1A). The pIPNs’ ESR values are comparable to the ESR of the AA100 sample and higher when compared to the neat PAAM (ESR of AA0~18). Nevertheless, such an ESR value is comparatively low for super absorbents such as PAA and PAAM. This could be because, at pH~6 (the swelling experiment is performed in distilled water), the prevailing number of the carboxylic groups of PAA are protonated, and ~40% of the carboxylic groups of PAA are in their anionic form [19]. Thus, two effects take place during water absorbance: (i) hydrogen bonds are formed either between –COOH groups of PAA [20] or between –COOH (from PAA) and –CONH_2_ groups (from PAAM), which implies additional constraints on the pIPNs’ swelling ability and explains the not very high ESR values; and (ii) the ionized carboxylic groups start to repulse each other and, the more carboxylic anions are formed, the stronger the repulsion, which results in the hydrogel expansion. This process lies behind the super absorbency of PAA. Thus, as the two effects act in opposite directions, no clear dependence of ESR on the pIPNs hydrogels’ composition is observed (also confirmed by the applied ANOVA analysis, Table S1).

The in situ formation of magnetite particles in the IPNs results in a significant increase in the ESR and the obtained pIPNs/magnetite composites swell much more than the neat IPNs (Figure 1B). Moreover, a clear dependence of ESR on the IPN composition is observed: the ESR decreases as the AA content decreases (Figure 1B), as also confirmed by ANOVA (Table S2 and Figures S1 and S2, Supplementary Information). This trend could be explained by the disruption of the hydrogen bonds in the neat pIPNs, due to the preferable interaction of -COOH groups from AA with Fe ions, as revealed by the ATR-IR spectra of neat IPNs (Figure S3). Thus, the secondary physical network in pIPNs is destroyed and the total network density is reduced, i.e., the swelling degree increases. In the pIPNs/magnetite composites, the carboxylic anions dictate their swelling behavior due to the repulsive interactions between adjacent -COO^−^ anions.

We have also detected an additional ionization of the pIPNs/magnetite composites due to the PAAM alkaline hydrolysis which takes place under the experimental conditions used for magnetite formation, namely pIPNs’ treatment with 6 M NaOH for 72 h (Figure S5):

−CONH_2_ + NaOH → −COO^−^ Na^+^ + NH_3_ (1)

In this way, some of the AAM monomeric units are transformed into sodium acrylate and this is confirmed when comparing the IR spectra of AA0 and AA0N (Figure S5A, Supplementary Information). Thus, the procedure for the iron oxide formation increased the number of -COO^−^ anions (Table S3, Figure S5) resulting in an enhanced electrostatic repulsion between adjacent chains and contributing to the increased ESR of pIPNs/magnetite composites compared to that of the neat pIPNs. The ESR of pIPNs/magnetite composites increases at higher PAA content, which is expected due to the enhanced number of COO- anions and the repulsion between adjacent anions in the pIPNs, resulting in both cases in a higher swelling degree.

When comparing the ESR of pIPNs/magnetite composites obtained using different concentrations of the Fe solutions used for the in situ iron oxide formation (namely X, Y and Z series in Materials and methods section), it is clearly seen that they do not differ in their ESR, i.e., the Fe ion concentration does not influence the ESR of pIPNs (Figure S6).

### 2.2. Number Average Molecular Mass between Crosslinks and Mesh Size of Neat pIPNs

As can be seen in Table 1, the polymer volume fraction $\nu_{2,s}$ in the swollen state increases as the AA content decreases, while the number average molecular mass between crosslinks decreases. This means that the network becomes denser as the PAA content decreases, which is in line with the ESR study of the pIPNs/magnetite composites (Figure 1B). The causes, as outlined above, are the interlaced IPN structure as well as the decrease in the number of charged groups (coming from PAA) that repulse each other and expand the polymer hydrogel upon swelling.

**Figure 2:**
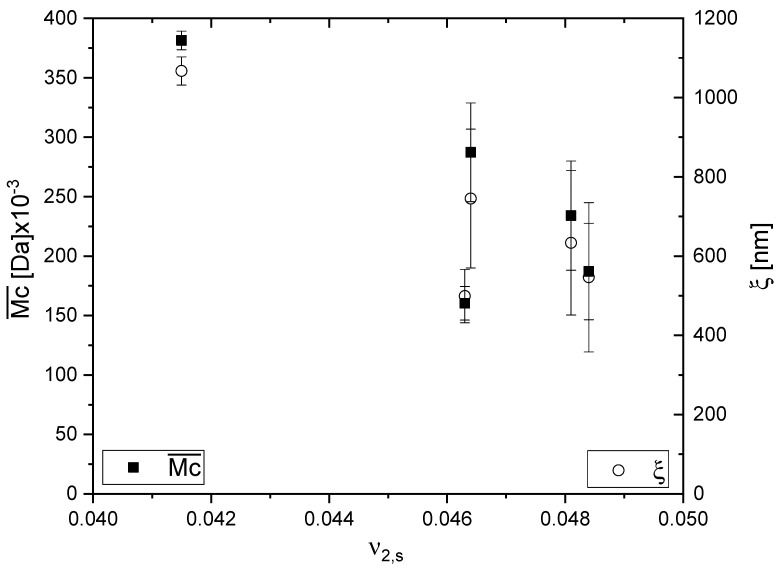
Dependence of the number average molecular mass between crosslinks and the mesh size of pIPNs hydrogels on polymer volume fraction.

The mesh size characterizes the space between macromolecular chains, and this is the space where a solute in a network could travel as well as being the space where iron oxide nanoparticles could be formed. That is why the mesh size is expected to play an important role in the in situ formation of iron oxides. Figure 2 presents the dependence of both the number average molecular mass between crosslinks as well as the mesh size of pIPNs hydrogels on the polymer volume fraction. As the polymer volume fraction, $\nu_{2,s},$ increases, both Mc and ξ decrease, which again proves the network density increase. This observation is in line with similar results for polymer networks reported elsewhere [21].

The theoretical number average molecular mass between crosslinks for pIPNs hydrogels, ${\bar{\;M}}_{c,t}$, was calculated (Equation (7)) and is presented in Table 1. Both Mc values show the same dependence on pIPNs’ composition, but they significantly differ in their values. The theoretically predicted ${\bar{\;M}}_{c,t}$ is much lower than the corresponding experimentally determined $\bar{\mathrm{Mc}}$ value. This can be explained by the fact that the theoretical predictions do not consider the presence of the linear PAAM chains which contribute to the overall pIPNs samples’ behavior, in particular their swelling. Following the model of Andrews et al. [22], the molecular network in a rubber sample is a dual or “hybrid” network, containing two types of chains: (1) chains which are at equilibrium when the sample is at its unstretched length (in our case these are the PAAM linear chains, part of the pIPN structure); and (2) chains which are at equilibrium when the sample is at its stretched length (this is the copolymer network). The interchain entanglements between the copolymer network and the linear polymers could possibly be the source of the significant difference between the two Mc values.

### 2.3. Determination of the Iron Content in pIPNs/Magnetite Composites

**Figure 3:**
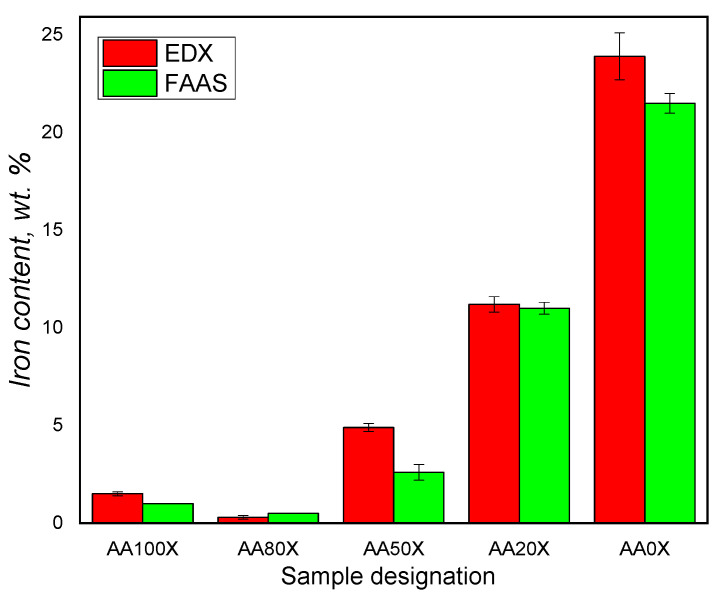
Iron ions content in pIPNs composites as determined by flame atomic absorption spectroscopy (FAAS) and energy-dispersive X-ray analysis (EDX).

**Figure 4:**
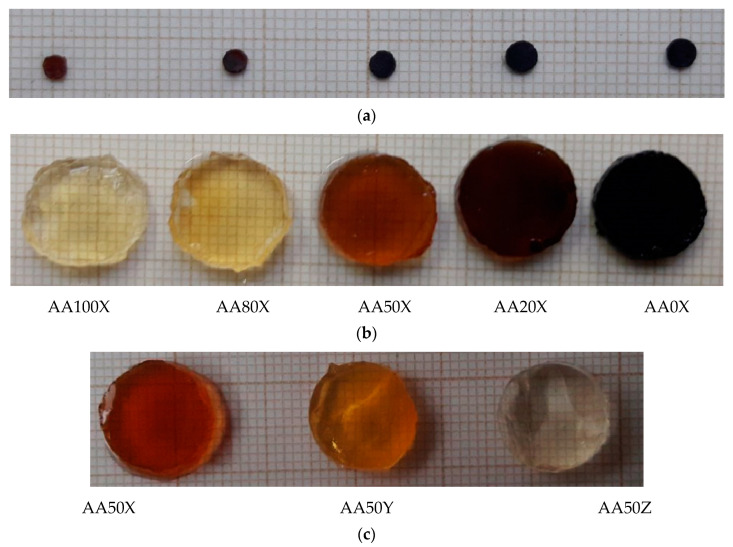
Appearance of the pIPNs/magnetite composites (**a**) when dry and (**b**) in their swollen state. (**c**) Comparison in the appearance of the AA50 composites obtained by swelling in Fe^3+^/Fe^2+^ solutions with different concentrations (Z denotes the lowest Fe ions concentration used).

The swelling ability of the pIPNs is expected to influence the quantity of iron ions that they absorb. As the amount of PAA in the pIPNs decreases, the absorbed iron increases, as revealed by two independent methods, namely flame atomic absorption spectroscopy (FAAS) and energy-dispersive X-ray analysis (EDX) (Figure 3). These results are supported by the visual appearance of the pIPNs composite hydrogels (Figure 4). The pIPNs hydrogels’ color changes (deepens) from transparent through yellowish to black as PAA content in the pIPNs decreases from AA100 to AA0. Thus, the pIPNs’ composition is a tool for controlling the in situ formation of magnetite within the P(AA-co-AAM)/PAAM pIPNs hydrogels. The quantity of absorbed Fe ions and hence the quantity of iron oxides formed in situ also influenced the crystallinity of the pIPNs composites. We have demonstrated that using different concentrations of the initial Fe^3+^/Fe^2+^ solution, changing in this way the amount of in situ-formed iron oxide, changed not only the visual appearance of the samples (Figure 4c) but also the crystallinity of the formed iron oxide (Figure S9).

The higher amount of Fe in the pIPNs with the lowest PAA content could be explained by the increased amount of in situ-formed (precipitated) iron oxide particles in these pIPNs.

The precipitation of the iron oxide in AA monomeric units-rich hydrogels is hampered by the complex formation between COO^−^ anions from PAA and Fe cations from the salts, which is confirmed by comparing the IR spectra of AA100 (i.e., neat PAA) and one of its composites, i.e., AA100Fe (Table S4, Figure S5B). It illustrates that the bands for −C=O antisym and ν_C=O_ –COOH in pIPNs observed in the spectrum of the PAA homopolymer (AA100) at 1700 cm^−1^ and 1653 cm^−1^ shift, respectively, to 1711 cm^−1^ and 1666 cm^−1^ in AA100Fe (which is the same pIPN sample swollen in Fe salts solution) due to fact that Fe ions interact preferably with –COO^-^ groups of PAA. In contrast, the amide I, II and III bands do not change their position after swelling in Fe solution, which is clearly seen when comparing the spectra of AA0 and AA0Fe, i.e., the same network after its swelling in Fe salts aqueous solution (Table S3, Figure S5A). Thus, the interaction between Fe ions and COO^−^ groups of PAA hampers the in situ formation of iron oxide particles. In contrast, in PAAM-rich pIPNs, the Fe ions are “free” to form iron oxide particles as the Fe ions do not interact with –CONH_2_ groups of PAAM (Figure S5A) and thus the quantity of iron oxides formed increases.

### 2.4. X-ray Diffraction of the pIPNs Composites

**Figure 5:**
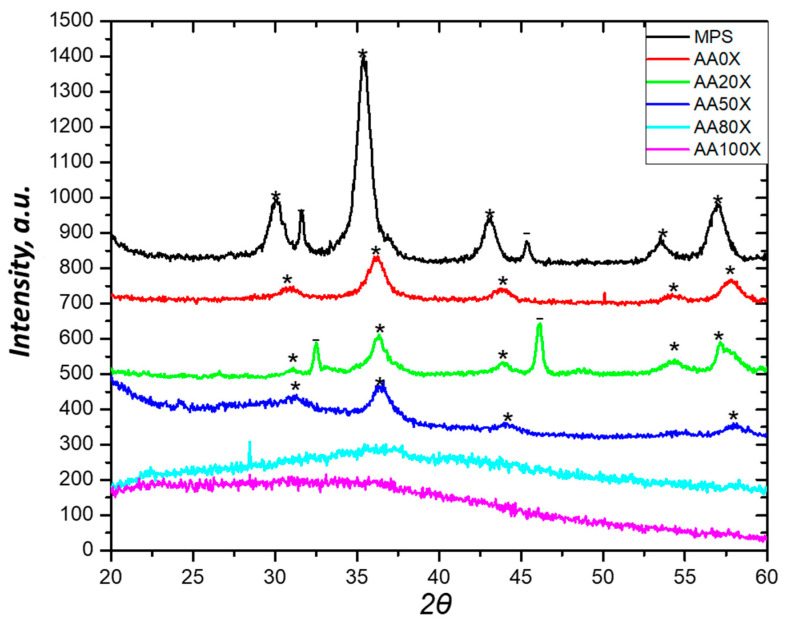
XRD diffractograms of pIPNs/magnetite composites.

*X*-ray diffraction was used to characterize the pIPNs iron oxides obtained in situ (Figure 5). The diffractograms of the pIPNs hydrogels with in situ-formed iron oxide particles show peaks which are characteristic of magnetite (at 2θ = 30.15; 35.4; 43.05; 53.5; and 57.0, marked with asterisk * in Figure 5) [23]. The hydrogels’ composition, i.e., the PAA/PAAM ratio, strongly influences the magnetite formation; in the samples where AA monomeric units prevail (AA100X and AA80X), no crystal peaks are detected, while in the samples AA50X, AA20X and AA0X, the magnetite peaks are clearly seen (Figure 5). This observation is in line with the explanation provided above of how the pIPNs’ composition determines the iron content in the respective pIPNs/magnetite composites. As mentioned above, the -COO^−^ groups’ interaction with Fe ions does not allow the formation of crystalline magnetite: the higher the COOH groups content, the lower the amount of crystalline magnetite.

For the sake of comparison, the X-ray diffractogram of neat iron oxide obtained using the same procedure for in situ preparation of the pIPNs/magnetite composites (designated as MPS in Table 2) is presented in Figure 5. The neat iron oxide (MPS sample) shows slightly shifted peak positions when compared to magnetite (PDF# 01-1111); these are most probably due to irregularities of the samples’ surface.

The crystallite size of magnetite particles was calculated using the peaks at 2θ = 35.4 D_hkl_(311) and 2θ = 43 D_hkl_(400) (Table 4). The magnetite crystallites are very small (≤ 11 nm) which explains well the peak broadening observed in their X-ray diffractograms (Figure 5). Thus, according to the XRD results, magnetite crystallites with size ≤11 nm form bigger polycrystalline particles (which we call magnetite particles in the manuscript).

In Table 4, the size of MPS magnetite crystallites is also provided for the sake of comparison. As could be expected, it is higher than the crystallite size of magnetite particles formed in situ in pIPNs due to the constraints that the hydrogel exerts on the magnetite formation.

**Figure 6:**
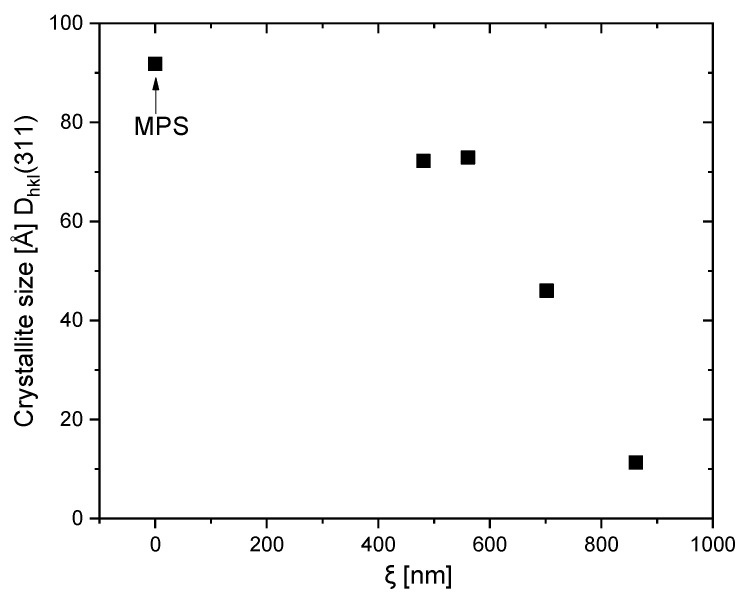
Dependence of the magnetite crystallite size on the pIPNs’ mesh size for pIPNs/magnetite composites.

Magnetite crystallite values, such as those obtained within this study, were reported when in situ precipitation of iron oxide in a neat PAAM network was performed. Specifically, 6 and 9 nm are reported by Sivudu et al. while, without the influence of any polymer, magnetite crystallites of size ~10 nm was observed [7]. This opens up the avenue for successful exploration of pIPNs as a template for the in situ formation of magnetite as a method for the manufacture of magnetite–polymer nanocomposites. Such a method may avoid the agglomeration of magnetite nanoparticles, which usually takes place when such nanocomposites are obtained via direct blending of the polymer and the ferric oxide particles [24]. Furthermore, the size and crystallinity of magnetite particles could be controlled via pIPNs’ composition as Figure 6 reveals: there is a clear relationship between the mesh size of the pIPNs and the crystallite size of the in situ-formed magnetite.

Moreover, the concentration of the initial Fe^3+^/Fe^2+^ solution could also be used to influence the quantity of the in situ-formed iron oxides as well as their crystallinity (Figure S9). The dilution of the Fe salts solution results in an insufficient quantity of Fe ions which could be further effectively involved in the formation of crystal phases and, thus, the crystallinity could be varied.

### 2.5. pH-Responsive Behavior of pIPNs and the pIPNs/Magnetite Composites

**Figure 7:**
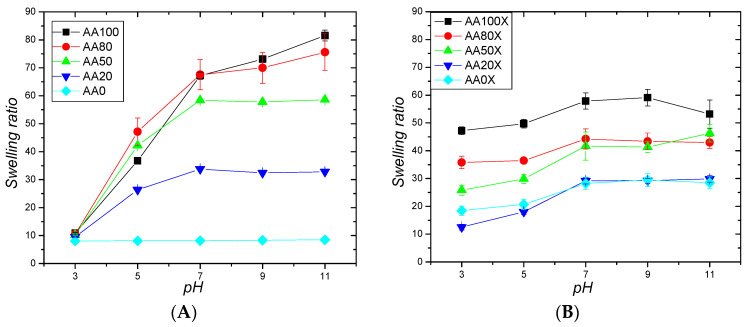
pH responsiveness of (**A**) neat pIPN hydrogels and (**B**) pIPNs/magnetite composite hydrogels.

The presence of –COOH groups in the pIPNs defines their ability to respond to changes in pH. At pH > pK_a_^COOH^ (pK_a_^COOH^ of PAA is in the range from 4.25 to 4.75) [25,26], –COOH groups are deprotonated, which results in negatively charged pIPNs. Thus, an electrostatic repulsion arises between neighboring chains and the hydrogel expands, leading to an ESR increase. This pH responsiveness is clearly seen for neat P(AA-co-AAM)/PAAM hydrogels in Figure 7A where:

- At pH = 3 (i.e., below pKa of COOH), all –COOH groups are protonated and the pIPNs hydrogels show a swelling ratio ~10 for all pIPNs compositions, i.e., AA content does not influence the ESR.
- At pH = 5 and above (i.e., above pKa of COOH), the polyanionic character of the pIPNs hydrogels makes the hydrogels increase their swelling ratio up to 70–80, i.e., 7 to 8 times, at an alkaline pH when compared to the swelling ratio obtained for pH = 3 (Figure 7A, Figure S10).

The ESR value is highly dependent on the hydrogel composition: the higher the AA content, the higher the ESR. As expected, the PAAM/PAAM IPN hydrogel (AA0 sample) does not show pH responsiveness. Thus, the AA/AAM monomeric units’ ratio in their copolymer is also a key tool for controlling the smart behavior of the P(AA-co-AAM)/PAAM hydrogels upon pH change.

The pIPNs/magnetite composite hydrogels also demonstrate pH responsiveness although their swelling ratio, for some compositions, increases “only” twice upon pH increase (Figure 7B). Several factors define the weaker effect of pH on the swelling ratio of pIPNs/magnetite composites. Firstly, magnetite particles act as additional crosslinking junctions, reducing the swelling ratio of the composite hydrogels in comparison to the neat pIPNs; this is clearly seen at higher pH when all COOH groups are deprotonated. Similar observations are reported for PAAM/magnetite composites where the swelling of magnetite-loaded PAAM hydrogels in physiological fluid was observed to decrease when increasing the amount of magnetite in the gel [12]. Secondly, the increased ionic strength of the media, as the pH value is adjusted using 0.1 M phosphate buffer and 0.1 M NaOH, is known to suppress the swelling of polyelectrolytes (in this particular case—PAA). Thirdly, the complexation between the carboxyl groups of PAA and iron ions partially shields the repulsion between adjacent COO^-^ thus diminishing the pH influence on the swelling ratio of pIPNs/magnetite composite hydrogels [27].

The different behavior of the AA0 sample (PAAM/PAAM pIPNs) and the respective composite AA0X (PAAM/PAAM pIPNs with in situ-formed magnetite) should be noted. While, as expected, AA0 shows no pH responsiveness (Figure 6A), the respective composite hydrogel exhibits some influence of pH on its swelling (Figure 6B). Similar results are reported in the literature for PAAM hydrogel loaded with magnetite nanoparticles; they also demonstrated pH responsiveness [6]. The authors explained the observed behavior by enhanced electrostatic forces between the amide groups and iron oxide. However, according to our results (Table S4, Figure S5A), which are further confirmed by a recent paper [13], this pH responsiveness could be due to the PAAM hydrolysis to PAA (Figure S4) which takes place during the magnetite formation where a high concentration of NaOH is used. Thus, COO^−^ anions appear along the PAAM chains which define the observed pH responsiveness of the respective composites.

### 2.6. Salt-Concentration Responsiveness of pIPNs and the pIPNs/Magnetite Composites

**Figure 8:**
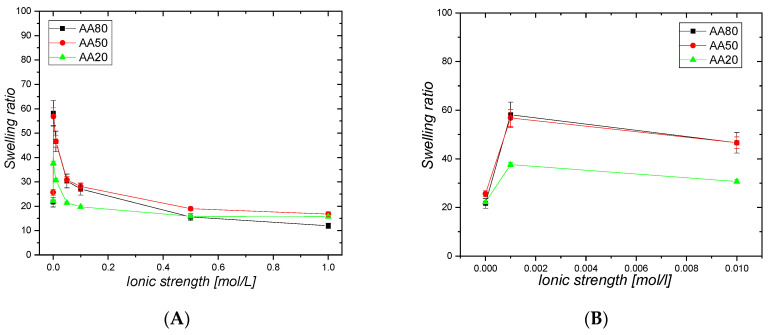

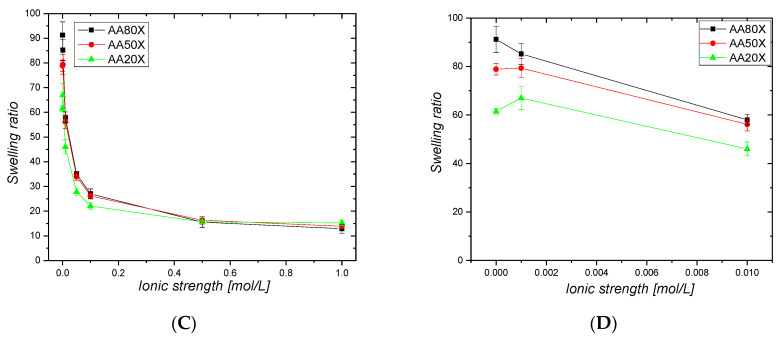
Ionic-strength responsiveness of pIPNs hydrogels: (**A**) and (**B**) without magnetite; (**C**) and (**D**) with in situ-formed magnetite. Figure (**B**) and (**D**) are provided in order to better illustrate the swelling ratio at ionic strength lower than 0.01 mol/L.

All pIPNs hydrogels—neat and composite ones—exhibit a polyelectrolyte effect, i.e., a decrease in their swelling ratio upon increasing the ionic strength of the medium (Figure 8). It is interesting to note the sharp increase in the swelling ratio (SR) of the neat pIPNs hydrogels at very low (0.001 M) NaCl concentration (Figure 8B, Table 3). The effect could be explained by the partial or complete destruction of the hydrogen bonds formed between PAA and PAAM by the stronger interaction of COOH groups with the sodium ions from NaCl. This results in liberation of part of the pIPNs chains and thus the SR sharply increases. The further increase in the ionic strength, however, shields up the repulsive interactions between carboxylic anions in the PAA and, correspondingly, the SR decreases.

For pIPNs/magnetite composite hydrogels, such SR increase at low NaCl concentrations is not observed (Figure 8C, samples with “X” in Table 3). Their SR decreases as the NaCl concentration increases most probably due to the lower number of hydrogen bonds between PAA and PAAM: the concurrent interaction of the PAA –COOH groups with Fe ions results in weaker PAA-PAAM interactions. The smaller number of hydrogen bonds in pIPNs/magnetite composites means a lower number of H-bonds that could be disrupted upon the addition of electrolytes and the resulting effect of the salt on the SR is weaker. This observation indirectly confirms the proposed explanation for the neat pIPNs’ swelling behavior at low NaCl concentration (Figure 8A). Moreover, due to the PAAM hydrolysis, the number of CONH_2_ groups decreases as they are transformed into sodium acrylates under the experimental conditions used for magnetite formation, which could also result in a decrease in the number of PAA-PAAM hydrogen bonds.

### 2.7. Temperature Responsiveness of pIPNs and pIPNs/Magnetite Composites

**Figure 9:**
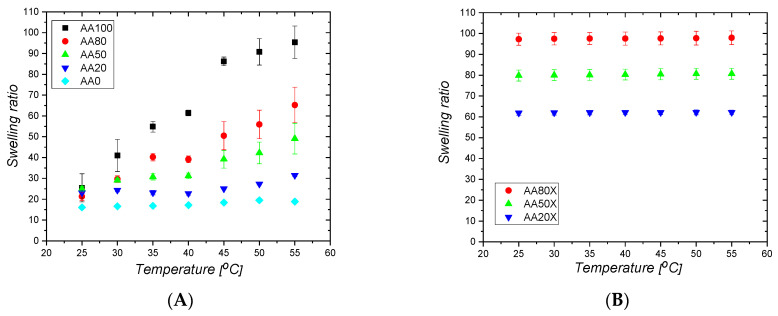
Temperature responsiveness of (**A**) neat P(AA-co-AAM)/PAAM pIPN and (**B**) pIPNs/magnetite hydrogels.

The hydrogen bonds between PAA and PAAM in the pIPNs define temperature responsiveness and PAA/PAAM-based materials show upper critical solution temperature behavior (UCST). The neat pIPNs hydrogels exhibit temperature responsiveness, which is influenced by their composition (Figure 9A): as the PAA content decreases, the PAAM content increases correspondingly, and the temperature responsiveness diminishes. The reason for this is the decreased number of inter- and intramolecular hydrogen bonds that results from variation of pIPNs composition, which defines less pronounced temperature responsiveness (Figure 9A).

The pIPNs/magnetite composites, however, do not show temperature responsiveness; their swelling ratio does not change upon temperature increase (Figure 9B). This confirms the explanation above that, due to the concurrent interaction of –COOH groups from PAA with Fe ions, the number of hydrogen bonds between –COOH and –CONH_2_ groups (from PAAM) is reduced and this results in diminishment of the temperature response. Moreover, the procedure for magnetite formation results in –CONH_2_ groups hydrolysis, i.e., a decrease in the number of sites where hydrogen bonds could be formed, additionally reducing the number of hydrogen bonds in the composites. All these factors result in the loss of temperature responsiveness in the pIPNs/magnetite composites (Figure 9B). Similar studies with hybrid pIPNs/magnetite materials obtained at lower Fe ions concentration (Figure S11) confirm this conclusion and reveal that even low Fe concentrations could make the pIPNs lose their temperature responsiveness.

### 2.8. Thermal Properties

**Figure 10:**
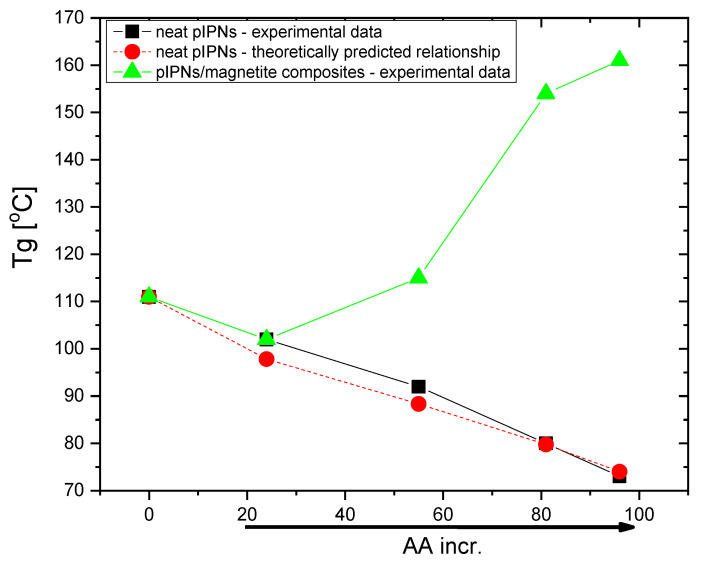
Dependence of pIPNs’ Tg on the PAA content for: (■) neat pIPNs—experimental data; (●) neat pIPNs—theoretically predicted relationship using the additivity law; and (▲) pIPNs/magnetite composites.

The thermal properties of neat pIPNs as well as of their composites containing magnetite were studied to evaluate the influence of magnetite on the pIPNs’ properties (Figures S12 and S13). The pIPNs show one Tg which is between the Tg’s of the neat PAA, namely 73 °C for the AA100 sample, and 111 °C for the neat AA0 sample (Table S6, Figure S12). The experimentally determined Tg (black squares) show a slight positive deviation from the dependence predicted by the additivity law (red dots), which could be explained by the hydrogen bonding between PAA and PAAM (Figure 10).

In contrast, for pIPNs/magnetite composites, Tg increases as PAA content increases (Table S6, Figure 10). As the ATR-IR data have shown, there is an interaction between COO^−^ anions and Fe^n+^ ions (Table S4), which imposes additional conformational constraints on the polymer segments involved in the binding of Fe ions. The reduction of the polymer flexibility is accompanied by an increase in Tg as reported for e.g., poly(ethylene oxide) interaction with Na^+^ and Li^+^ ions [28]. The authors report an exponential increase in Tg as salt concentration increases, which enhances the polymer–metal ion interactions. The case here is similar: the number of interactions between PAA and Fe ions increases as PAA content increases (Figure 10).

The Tg values for AA0X and AA20X do not show any deviation from the respective neat pIPNs, which could mean that, once formed, the magnetite does not strongly interact with the polymer network, which is also revealed by ATR-IR (Figure S5, Table S4). Thus, the positive deviation in the Tg dependence on PAA content is related mainly to the interaction between –COO^−^ pendant groups and Fe ions [29].

### 2.9. Scanning Electron Microscopy

**Figure 11:**
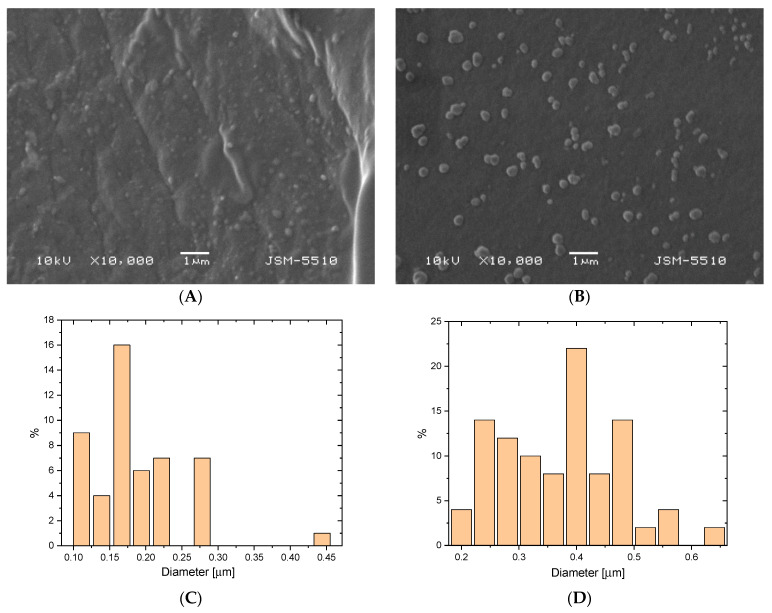
Morphology and magnetite particles’ size distribution in pIPNs/magnetite composites for: AA80X (**A**) and (**C**); AA20X (**B**) and (**D**).

The morphology of the broken surface of two pIPNs/magnetite composites are presented in Figure 11. Both samples were chosen as they present two border cases: AA80X has a lower iron content than AA20X. Moreover, the latter is proven to contain magnetite rather than Fe ions (Figure 5). The in situ-formed magnetite particles in AA80X could be clearly seen as bright spots that are evenly dispersed within the polymer matrix (Figure 11A). The SEM image of the AA20X sample reveals a greater size of the formed magnetite particles (Figure 11B) when compared to the particles seen in AA80X. The average size of magnetite particles in AA80X is ~200 nm (Figure 11C) while in AA20X they have an average diameter of ~400 nm (Figure 11D). These results are well aligned with the XRD data and confirm the conclusion that the copolymer composition controls the process of in situ iron oxide formation. Moreover, the smaller mesh size of the pIPNs provides a greater saturation in its loops, leading to the formation of magnetite particles with higher crystallinity.

### 2.10. Magnetic Properties

**Figure 12:**
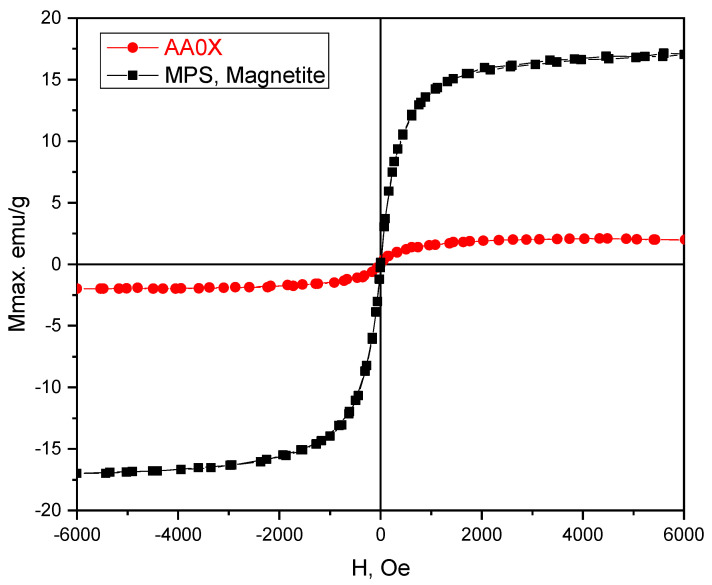
Magnetization of neat MPS and PAAM/PAAM pIPNs/magnetite composites.

The magnetic properties of pure magnetite (MPS) and the pIPNs/magnetite composites were studied using a vibrating sample magnetometer (VSM) at room temperature (Figure 12).

The magnetization response, i.e., magnetization versus the applied magnetic field, has the well-known S-shaped curve, with coercive field and remanent magnetization very close (within the experimental error) to zero. Thus, both the pure magnetite (MPS) as well as magnetite/pIPNs composite hydrogels exhibit superparamagnetic behavior, i.e., they become magnetized when a magnetic field is applied but have no permanent magnetization (remanence) after the magnetic field is removed. It is clearly understood from the literature that magnetite nanoparticles with particle sizes of less than 20 nm are superparamagnetic and single domain, since 20 nm is less than the domain size for that material [30]. The magnetization values for iron oxide nanoparticles are usually between 30 and 50 emu/g [31], while higher values (e.g., 90 emu/g) have been observed for bulk materials. Here, the lower values for AA0X magnetization (~3 emu/g) could be related mainly to the small particle sizes (less than 20 nm), as confirmed by SEM (Figure 11). The factors which are known to contribute to the magnetization value of superparamagnetic iron oxide nanoparticles, are (i) the size of the particles; (ii) the spacing between the nanoparticles (coatings such as silica or polymers separate the magnetic domains, allowing each individual magnetite particle to act independently and thus enhancing the net magnetism per gram); and (iii) the crystalline structure of the iron oxide [31]. The size and the crystallinity are the most probable factors contributing to the lower magnetization of MPS as compared to that of similar particles reported in the literature.

The saturation magnetization of the hybrid hydrogel at room temperature is significantly lower than that of the pure magnetite samples. The cause is the diamagnetic contribution of the polymer matrix within which the magnetite nanoparticles are evenly dispersed, the mass of which was not possible to estimate and subtract.

## 3. Conclusions

This study reveals the synthesis of novel P(AA-co-AAM)/PAAM pIPNs and their composites with magnetite, obtained via in situ deposition. Formation of magnetite particles takes place via a complex mechanism involving the predominant role of PAA as an Fe^2+^/Fe^3+^ binding component. The results demonstrate that the pIPNs’ composition is a powerful tool to control the size, crystallinity and quantity of the magnetite particles deposited in situ. SEM and XRD studies clearly confirm these conclusions. Moreover, the magnetite nanoparticles formed in situ act as additional crosslinks for the pIPNs networks, resulting in decreased swelling as well as reduced pH and ionic strength responsiveness of the pIPNs/magnetite composites when compared to the neat pIPNs. The valuation of the magnetic properties of the obtained composite hydrogels reveals their superparamagnetic properties with magnetization of ~3 emu/g. The results are encouraging and could be further used to widen the application of IPNs, in particular pIPNs, as templates for the preparation of polymer nanocomposites. Such novel inorganic/organic composites could find a wide range of applications as smart systems with defined pH and salt concentration responsiveness.

## 4. Materials and Methods

### 4.1. Materials

Iron (III) chloride, anhydrous and cyclohexane were purchased from Fisher Chemicals (Fisher Scientific, Waltham, MA, USA). Iron (II) sulfate heptahydrate, acrylic acid, acrylamide, potassium persulfate, N, N’-methylene-bis-acrylamide, N, N, N’, N’-tetramethylethylene diamine (TEMED), sodium hydroxide, nitric acid, perchloric acid and phosphoric acid were purchased from Sigma Aldrich, USA. Polyacrylamide (PAAM) (Mw > 5,000,000) was purchased from BDH Laboratory reagents, Poole, England. All reagents were used as received.

### 4.2. Methods

#### 4.2.1. Synthesis of P(AA-co-AAM)/PAAM pIPNs

P(AA-co-AAM)/PAAM pIPNs were synthesized by free radical polymerization. In summary, a defined amount of each monomer (Table 4) was dissolved in 1 wt% aqueous solution of linear PAAM (Mw = 5,000,000) giving a final total monomer concentration of 1.4 M. The initiator potassium persulfate (0.3 mol% to the total monomer content) and the crosslinking agent, N, N’-methylene-bis-acrylamide (2 mol% to the total monomer content) were added and the solution was homogenized using a magnetic stirrer at 350 rpm until all components were fully dissolved. Next, TEMED (0.2 *v*/*v*%) was added and the solution was further homogenized for 3 min. The obtained solution was placed between two glasses separated with a 1 mm thick rubber spacer and the polymerization took place over 45 min at 60 °C. After the end of the polymerization period, the obtained hydrogels were carefully removed from the glasses and placed in distilled water to purify them of any residuals. Water was changed 3 times daily until no residuals were found in the wastewater, as monitored by UV spectrophotometry. The purified hydrogels were cut into disk-shaped pieces and left to dry.

#### 4.2.2. P(AA-co-AAM) Composition

The ratio between AA and AAM monomeric units in their copolymer was estimated using the Mayo–Lewis equation:

(2) $$\frac{M_{\mathrm{AA}}^{P}}{M_{\mathrm{AM}}^{P}}=\frac{\left[\mathrm{AA}\right]\cdot \left(r_{1}\cdot \left[\mathrm{AA}\right]+\left[\mathrm{AM}\right]\right)}{\left[\mathrm{AM}\right]\cdot (\left[\mathrm{AA}+r_{2}\cdot \left[\mathrm{AM}\right]\right)}$$

where [AA] and [AM] are the molar concentrations of the monomers AA and AAM in the starting solution. $M_{\mathrm{AA}}^{P}$ and $M_{\mathrm{AM}}^{P}$ are the molar fractions of the corresponding monomeric units in the copolymer. r_1_ and r_2_ are, respectively, the reactivity ratios for the AA/AAM copolymerization. As the reactivity ratio depends on the pH of the reaction mixture, its values were taken at the closest point to the respective initial comonomer solution pH (Table 4) [32].

#### 4.2.3. Preparation of pIPNs/Magnetite Composite Hydrogels

An aqueous solution of two Fe salts, namely FeCl_3_ (0.30 M) and FeSO_4_.7H_2_O (0.15 M), was prepared and dry disk-shaped samples of pIPNs were immersed in it for 72 h. Fe^2+^/Fe^3+^ loaded pIPN hydrogels were taken out of the solution, washed with distilled water, and placed in 6 M NaOH for 72 h. The same procedure was repeated using two other concentrations of the mixed Fe^n+^ salts solutions: 10 times diluted, i.e., FeCl_3_ (0.03 M) and FeSO_4_.7H_2_O (0.015 M) (composite hydrogels designated with “Y”), and 100 times diluted, i.e., FeCl_3_ (0.003 M) and FeSO_4_.7H_2_O (0.0015 M) (composite hydrogels designated with “Z”), respectively. The obtained composite hydrogels were washed with distilled water for 7 days, the water being changed twice daily. All hydrogels were left to dry in ambient conditions. The compositions of the obtained composites are presented in Table 2.

AA100Fe and AA0Fe samples (Table 5) were obtained to evaluate the swelling in Fe^n+^ mixed salt aqueous solution of the neat PAA and PAAM, respectively. Similarly, AA100N and AA0N samples (Table 5) were obtained to evaluate the swelling in NaOH aqueous solution of the neat PAA and PAAM samples. Moreover, for the sake of comparison, neat magnetite particles were synthesized via co-precipitation. In brief, the aqueous solution of FeCl_3_(0.30 M) and FeSO_4_.7H_2_O (0.15 M) was alkalized with 6 M NaOH to reach pH = 11. The obtained black precipitate was magnetically decanted, washed five times with distilled water and dried under ambient conditions. These particles are designated as MPS in Table 5. Their size was found to be 388 ± 143 nm via DLS (Figure S16, Supplementary Information).

#### 4.2.4. Swelling Behavior

The volume of the dry polymer network, $V_{d}$, and the volume of the hydrogel at equilibrium swelling in water, $V_{s}$, were determined using the following equations:

(3) $$V_{d}=\frac{m_{d,a}-m_{d,h}}{\rho }$$

(4) $$V_{s}=\frac{m_{s,a}-m_{s,h}}{\rho }$$

Here m_d,a_ and m_d,h_ are the weights of the dry polymer networks measured in air and in cyclohexane, respectively, while m_s,a_ and m_s,h_ are the weights of the swollen hydrogels measured in air and in cyclohexane, respectively.

The polymer volume fraction, $\nu_{2,s}$, in the swollen state was calculated by using the equation:

(5) $$\nu_{2,s}=\frac{V_{d}}{V_{s}}$$

The number average molecular mass between crosslinks ($\bar{\mathrm{Mc}}$) was determined by the swelling experiments according to the Flory–Rehner equation:

(6) $$\frac{1}{\bar{\mathrm{Mc}}}=-\frac{\frac{\bar{\nu }}{V_{1}}[\ln\left(1-\nu_{2,s}\right)+\nu_{2,s}+{\chi \nu }_{2,s}^{2}]}{\left(\sqrt[{3}]{\nu_{2,s}}-0.5\nu_{2,s}\right)}$$

where $V_{1}$ is the molar volume of water (18 cm^3^/mol) [21], $\bar{\nu }$ is the specific volume of the polymer (0.67) [33], and χ is the Flory–Huggins interaction parameter between the polymer and water, which is 0.5 for PAA and 0.49 for PAAM [33].

The theoretical number average molecular mass between crosslinks, ${\bar{\;M}}_{c,t}$, was calculated by using the equation:

(7) $${\bar{\;M}}_{c,t}=\frac{M_{r}}{2X}$$

where $M_{r}$ is the molecular mass of the polymer repeat unit determined as weighted average and X is the nominal crosslinking ratio, calculated as the molar ratio between total monomer content and the crosslinking agent.

The network mesh size, ξ, was calculated using the equation:

(8) $$\xi =\frac{1}{\sqrt[{3}]{\nu_{2,s}}}\sqrt{\frac{2C_{n}\bar{\mathrm{Mc}}\;}{M_{r}}}l$$

Here $C_{n}$ is the Flory characteristic ratio ($C_{n}^{PAA}$ = 6.7 and $C_{n}^{PAAM}$ 2.72) [33,34], and l is the carbon–carbon bond length (1.54 Å).

#### 4.2.5. Equilibrium Swelling Ratio (ESR)

Both the pIPNs and the pIPNs/magnetite composites were left to swell in water until they reached a constant weight. Next, their equilibrium swelling ratio (ESR) was determined by the equation:

(9) $$\mathrm{ESR}=\frac{m_{\mathrm{sw}}-m_{\mathrm{dry}}}{m_{\mathrm{dry}}}$$

where $m_{\mathrm{sw}}$ and $m_{\mathrm{dry}}$ denote the weights of the sample in swollen state and dry state, respectively. pH of water used in the experiment was measured to be ~5.6 (Hanna instruments HI 2211 pH/ORP meter).

#### 4.2.6. pH Responsive Behavior of pIPNs and pIPNs/Magnetite Hydrogels

The pH responsiveness of pIPNs and the pIPNs/magnetite composite hydrogels were determined in the pH range from 3 to 11 at 25 ± 1 °C. Briefly, dry disk-shaped samples (4.5 mm diameter) were swollen in buffer solution with defined pH until they reached a constant weight (for ~24 h). The swelling ratio at the respective pH (SR^pH^) was calculated by using the equation:

(10) $${\mathrm{SR}}^{\mathrm{pH}}=\frac{m_{\mathrm{sw}}^{\mathrm{pH}}-m_{\mathrm{dry}}}{m_{\mathrm{dry}}}$$

where $m_{\mathrm{sw}}^{\mathrm{pH}}$ and $m_{\mathrm{dry}}$ denote, respectively, the weights of the sample in its swollen state at certain pH and in its dry state. The buffer solutions were prepared following a procedure described in the literature [35].

#### 4.2.7. Temperature Responsive Behavior of pIPNs and the pIPNs/Magnetite Composite Hydrogels

The temperature responsiveness of pIPNs and their magnetite composites was tested in the temperature range from 25 to 55 °C in water. Dry disk-shaped samples (4.5 mm diameter) were swollen in water at a defined temperature for 8 h. The swelling ratio at the defined temperature (SR^Temp^) was calculated by using the equation:

(11) $${\mathrm{SR}}^{\mathrm{Temp}}=\frac{m_{\mathrm{sw}}^{\mathrm{Temp}}-m_{\mathrm{dry}}}{m_{\mathrm{dry}}}$$

where $m_{\mathrm{sw}}^{\mathrm{Temp}}$ and $m_{\mathrm{dry}}$ denote the weight of the sample in its swollen state at certain temperature and in its dry state, respectively.

#### 4.2.8. Ionic Strength Responsive Behavior of pIPNs and pIPNs/Magnetite Composite Hydrogels

The ionic strength responsiveness of pIPNs and the pIPNs/magnetite composites was measured in aqueous NaCl solutions with concentration ranging from 0.001 M to 1 M as well as in pure water (0 M). In brief, dry disk-shaped samples (4.5 mm diameter) were swollen in a solution with certain NaCl concentration until they reached a constant weight (~24 h). The swelling ratio (SR^I^) was calculated for each NaCl concentration by using the equation:

(12) $${\mathrm{SR}}^{I}=\frac{m_{\mathrm{sw}}^{I}-m_{\mathrm{dry}}}{m_{\mathrm{dry}}}$$

where $m_{\mathrm{sw}}^{I}$ and $m_{\mathrm{dry}}$ denote the weights of the sample in its swollen state at certain NaCl concentration and in its dry state, respectively.

#### 4.2.9. Attenuated Total Reflectance-FTIR (ATR-FTIR)

pIPNs and the pIPNs/magnetite composites were characterized using infrared spectroscopy (IR) in a regime of attenuated total reflectance by using an IRAffinity-1 Shimadzu Fourier transform infrared (FTIR) spectrophotometer with MIRacle attenuated total reflectance attachment. The samples were studied as dry solids, without any preliminary treatment.

#### 4.2.10. Differential Scanning Calorimetry (DSC)

Differential scanning calorimetry tests were performed on DSC apparatus Q200, TA Instruments, USA. Dry samples (~5–6 mg) were heated from −50 to 150 °C, then cooled to −50 °C before being heated again to 230 °C at a 10 °C/min heating rate under nitrogen flow (50 mL/min). The results that were used and reported within the study are from the second heating run.

#### 4.2.11. X-ray Diffraction (XRD)

A Siemens D500 diffractometer, Germany, with secondary monochromator and Cu-K_α_ radiation was used to obtain the diffractograms over 2θ range of 10–80° with a step of 0.03° and count time of 10 s. The search-match program Match3 was used to identify the crystal phases. The crystallite size D_hkl_ (in Å) in direction perpendicular to the (hkl) planes (311) and (400) was calculated according to Scherrer’s formula:

(13) $$D_{\langle \mathrm{hkl}\rangle }=\frac{0.9*\lambda }{\beta_{\frac{1}{2}}\cos\theta }$$

where λ = 1.542 Å is the wavelength used, 2θ is the reflection position and β_1/2_, in rad, is the physical integral width of the reflection hkl positioned at 2θ. Gaussian function was used to approximate the reflections. β_1/2_ was obtained from the experimental width *B* and instrumental width b.

#### 4.2.12. Iron Content Determination in pIPNs/Magnetite Composites

##### Flame Atomic Absorption Spectroscopy

Pieces from each pIPNs/magnetite composite were weighed (~0.2–0.3 g) and immersed in 2 mL conc. nitric acid in a 25 mL beaker. The beaker was covered with watch glass and then heated on a hot plate at 90–100 °C for 1 hour. This time was enough to partially degrade the polymer. The solution was then cooled down to room temperature. Then, 1 mL conc. perchloric acid was added and the beaker was heated again to 100–120 °C until white fumes appeared, and complete polymer degradation was achieved. After cooling down, the solution was diluted with distilled water and transferred to a 25 mL volumetric flask. The concentration of Fe ions was determined by flame atomic absorption spectroscopy (Perkin Elmer Analyst 400, Waltham, MA, USA) using a calibrating curve (Figure S17, Supplementary Information).

##### Scanning Electron Microscopy (SEM) with Energy-Dispersive X-ray Spectroscopy (EDX)

To determine the iron content as well as the magnetite particles’ dispersion within the composites, the fractured surface of dry samples was covered with a thin carbon film (~10 nm). The samples were examined under a scanning electron microscope Lyra 3 XMU (Tescan), operating at 10 kV and coupled with EBSD and EDX analysis systems (Quantax 200, Bruker, Billerica, MA, USA).

##### Transmission Electron Microscopy (TEM)

Dry pIPNs/magnetite composites were cut into slices of ~100 nm width using an ultramicrotome (Leica EM UC7). The slices were examined under HR TEM (JEOL, JEM-2100), operating at 200 kV.

##### Magnetic Measurements

Magnetization curves were measured at room temperature using a vibrating sample magnetometer (VSM) in fields up to 6 kOe. The samples were prepared by pressing the powder into cylindrical (*Ø* = 3 mm, *h* = 10 mm) quartz containers so that the particles were unable to move during the measurements.

## Figures and Tables

**Table 1:** P(AA-co-AAM)/PAAM pIPNs hydrogels swelling characteristics in distilled water.

Sample Designation	$\nu_{2,s}$	$\bar{Mc}$ [Da]	${\bar{\;M}}_{c,t}$ [Da]	ξ [nm]
	Equation (5)	Equation (6)	Equation (7)	Equation (8)
AA100	0.0415 ± 0.0005	355,676 ± 11,809	1801	1144 ± 24
AA80	0.0464 ± 0.0037	248,403 ± 58,424	1797	862 ± 125
AA50	0.0481 ± 0.0069	211,178 ± 60,791	1790	702 ± 138
AA20	0.0484 ± 0.0059	182,124 ± 62,749	1783	561 ± 122
AA0	0.0463 ± 0.0028	166,366 ± 22,494	1777	481 ± 42

**Table 2:** Crystallite size of magnetite particles formed in situ in pIPNs at two 2θ values.

Sample	Crystallite Size [Å] at 2θ = 35.4 D_hkl_ (311)	Crystallite Size [Å] at 2θ ~ 43 D_hkl_ (400)
AA100X	amorphous	amorphous
AA80X	11	amorphous
AA50X	46	30
AA20X	73	96
AA0X	72	90
MPS	92	98

**Table 3:** Swelling ratios of pIPNs and pIPNs/magnetite composites in water and in 0.001 M NaCl aqueous solution.

Sample	H_2_O	0.001 M NaCl
AA80	22 ± 2	58 ± 5
AA80X	91 ± 5	85 ± 4
AA50	26 ± 1	57 ± 2
AA50X	79 ± 2	79 ± 4
AA20	22 ± 1	39 ± 1
AA20X	61 ± 1	67 ± 5

**Table 4:** Composition of the initial polymerization solutions as well as of the P(AA-co-AAM) copolymer (obtained by using the Mayo–Lewis equation).

Sample	Initial Polymerization Solution Composition			Reactivity Ratios Values		Copolymer Composition	
	AA [mol. %]	AAM [mol. %]	pH	r_1_	r_2_	AA * [mol. part]	AAM [mol. part]
AA100	100	0	2.7	PAA homopolymerization		0.96	-
AA80	80	20	2.9	1.34	0.69	0.81	0.19
AA50	50	50	3.3	1.34	0.69	0.55	0.45
AA20	20	80	3.7	1.28	0.82	0.24	0.76
AA0	0	100	6.2	PAAM homopolymerization		0	1

* N,N’-methylene-bis-acrylamide amount taken into account.

**Table 5:** pIPN hydrogels with in situ-formed magnetite particles via co-precipitation.

Sample Designation	AA [mol. %]	AAM [mol. %]	Fe^3+^ [mol/L]	Fe^2+^ [mol/L]	6 M NaOH
AA100X	100	0	0.30	0.15	yes
AA80X	80	20	0.30	0.15	yes
AA50X	50	50	0.30	0.15	yes
AA20X	20	80	0.30	0.15	yes
AA0X	0	100	0.003	0.0015	yes
10 times dilution					
AA80Y	80	20	0.03	0.015	yes
AA50Y	50	50	0.03	0.015	yes
AA20Y	20	80	0.03	0.015	yes
100 times dilution					
AA80Z	80	20	0.003	0.0015	yes
AA50Z	50	50	0.003	0.0015	yes
AA20Z	20	80	0.003	0.0015	yes
AA100Fe *	100	0	0.30	0.15	no
AA0Fe *	0	100	0.30	0.15	no
AA100N *	100	0	no	no	yes
AA0N *	0	100	no	no	yes
MPS *	-	-	0.003	0.0015	yes

* Samples obtained to be as referent.

## Data Availability

The raw/processed data required to reproduce these findings cannot be shared at this time as the data also form part of an ongoing study.

## Supplementary Materials

The following supporting information can be downloaded at: https://www.mdpi.com/article/10.3390/gels9050365/s1, [12,36,37,38,39,40,41] Table S1. ANOVA data for the dependence of ESR on the pIPNs compostion for neat IPNs, Table S2. An effect size of groups’ ESR. Generalized eta squared-ges of 0.945 (95%) means that 95% of the change in the ESR can be accounted for the treatment conditions. Figure S1. One-way single factor ANOVA results. Variance of ESR between groups. Figure S2. P.adjust dependence from Tukey test for significance. Figure S3. ATR-IR spectrum of neat pIPNs (A) and samples AA0 and AA20, showing the band shifting due to hydrogen bonding (B). Figure S4. ATR-IR spectra of the pIPNs, obtained with PAA homopolymer (AA100N) and PAAM hopomopolymer (AA0N) after treatment with 6 M NaOH for 72 h. Table S3. Band assignments the pIPNS IR spectra with homopolymer PAAM (AA0 sample). Table S4. Band assignments of the pIPNs with homopolymer PAA (AA100 sample). Figure S5. ATR-IR spectra of pIPNs (A) with homopolymer PAAM: neat (AA0); with absorbed Fe ions (AA0Fe); treated with 6M NaOH for 72 h (AA0N); and the respective magnetite/PAAM composite AA0X; and (B) with homopolymer PAA: neat (AA100); with absorbed Fe ions (AA100Fe); treated with 6M NaOH for 72 h (AA100N); and the respective magnetite/pIPNs composite (AA100X). Figure S6. Equilibrium swelling ratio of pIPNs hydrogels with in situ formed iron oxide obtained via using different Fe aqueous solutions concentration: X (Fe3+—0.30 mol/L; Fe2+—0.15 mol/L); Y (Fe3+—0.030 mol/L; Fe2+—0.015 mol/L) Z (Fe3+—0.003 mol/L; Fe2+—0.0015 mol/L). Table S5. ANOVA analysis on the dependence of initial iron solution on the ESR of the resulting hybrid hydrogels. Figure S7. Iron content as determined by Flame Atomic Absorption Spectroscopy (FAAS) in pIPNs composite materials with in situ formed iron oxide obtained via using different Fe aqueous solutions concentration: X (Fe3+—0.30 mol/L; Fe2+ —0.15 mol/L); Y (Fe3+—0.030 mol/L; Fe2+—0.015 mol/L) Z (Fe3+—0.003 mol/L; Fe2+—0.0015 mol/L). Figure S8. XRD patterns of sample AA20X in is dry and swollen state. Figure S9. XRD diffractograms of pIPNs/magnetite composites with different composition with in situ formed magnetite. Figure S10. Comparison between the swelling ratios of pIPNs and the pIPNs/magnetite composites at pH = 5. Figure S11. Temperature responsiveness of pIPNs/magnetite composite hydrogels. Figure S12. DSC thermograms of neat pIPNs (the 2nd heat scan from heat/cool/heat run is presented). Figure S13. DSC thermograms of pIPNs/magnetite composites (the 2nd heat scan from the heat/cool/heat run is presented). Table S6. Experimentally determined Tg of pIPNs and pIPNs/magnetite composites. Figure S14. ATR-IR spectrum of pIPNs/magnetite composites. It is clearly visible there is no significant difference between samples. Figure S15. Normalized concentration of TC removed by AA0X (A) and by of potassium persulfate activation (B). Figure S16. Size of the neat magnetite particles MPS as revealed by DLS. Figure S17. Calibration curve for Fen+ ions, used in FAAS experiments.
