# Supplementary material for: Poly(acrylic acid-co-acrylamide)/Polyacrylamide pIPNs/Magnetite Composite Hydrogels: Synthesis and Characterization
Source: Gels. 2023 Apr 26;9(5):365. doi: 10.3390/gels9050365 (PMC10217237; doi:10.3390/gels9050365)
Supplement: Supplementary file 1 [file gels-09-00365-s001.zip › gels-2306677-supplementary.pdf]

Article

Poly(Acrylic Acid-co-Acrylamide)/Polyacrylamide pIPNs/Magnetite Composite Hydrogels: Synthesis and Characterization

Marin Simeonov [1,*], Anton Atanasov Apostolov [1], Milena Georgieva [2], Dimitar Tzankov [2] and Elena Vassileva [1]

[1] Laboratory on Structure and Properties of Polymers, Faculty of Chemistry and Pharmacy, University of Sofia "St. Kliment Ohridski", 1, James Bourchier blvd., 1164 Sofia, Bulgaria
[2] Faculty of Physics, University of Sofia "St. Kliment Ohridski", 5, James Bourchier blvd., 1164 Sofia, Bulgaria
* Correspondence: m.simeonov@chem.uni-sofia.bg

Supplementary Information

Statistical analysis of the swelling data

ESR of neat pIPNs

The ESR values were analyzed by one-way single factor ANOVA. Since $F<F_{crit}$ and the p-value is ~0.3 ($p>>0.05$) (Table S1) the difference between ESR values is insignificant and no dependence of ESR on the copolymer composition is observed.

Table S1. ANOVA data for the dependence of ESR on the pIPNs compostion for neat IPNs

Source of Variation	*SS*	*df*	*MS*	*F*	*P-value*	F_{crit}
Between Groups	*170.8761*	*4*	*42.71902*	*1.38096*	*0.308259*	*3.47805*
Within Groups	*309.3429*	*10*	*30.93429*			
Total	*480.219*	*14*				

ESR of pIPNs/magnetite composites

The results were analyzed by one-way single factor ANOVA. Since $F>F_{crit}$ and the p-value is 2.8. 10^{-6} ($p<<0.05$) (Table S2), the difference in the ESR values is significant which means that there is a dependence of the ESR on the copolymer composition. The results were analyzed also with Tukey test to evaluate the differences between the different groups, i.e. the different copolymer compositions. The results are presented in Table S2 and Figures S1 and S2. The significant differences between groups are denoted with asterisks, the higher number of asterisks means higher difference. As it can be seen, there is a dependence of the ESR on the pIPNs/magnetite composition: the higher the amount of PAA is, the higher is the ESR.

ANOVA Table (type II tests)

Table S2. An effect size of groups' ESR. Generalized eta squared-ges of 0.945 (95%) means that 95% of the change in the ESR can be accounted for the treatment conditions.

Effect	DFn		DFd	F	p	p<0.05	ges
Group	4		10	43.223	2.8e-06	*	0.945

term	group1	group2	null.value	estimate	conf.low	conf.high	p.adj	p.adj.signif
1 group	AA100X	AA20X	0	-38.7	-50.2	-27.2	0.0000047	****
2 group	AA100X	AA0X	0	-28.2	-39.7	-16.7	0.0000817	****
3 group	AA100X	AA50X	0	-22.0	-33.5	-10.5	0.000654	***
4 group	AA100X	AA80X	0	-4.55	-16.0	6.92	0.694	ns
5 group	AA20X	AA0X	0	10.5	-0.953	22.0	0.0763	ns
6 group	AA20X	AA50X	0	16.7	5.26	28.2	0.00507	**
7 group	AA20X	AA80X	0	34.2	22.7	45.6	0.0000148	****
8 group	AA0X	AA50X	0	6.21	-5.27	17.7	0.434	ns
9 group	AA0X	AA80X	0	23.6	12.2	35.1	0.000363	***
10 group	AA50X	AA80X	0	17.4	5.95	28.9	0.00381	**

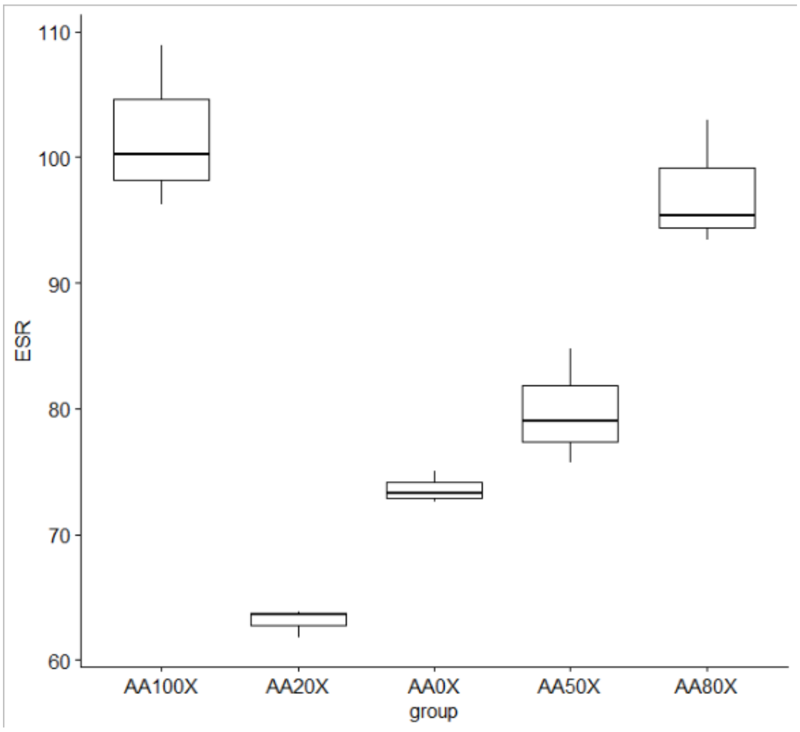

Figure S1. One-way single factor ANOVA results. Variance of ESR between groups.

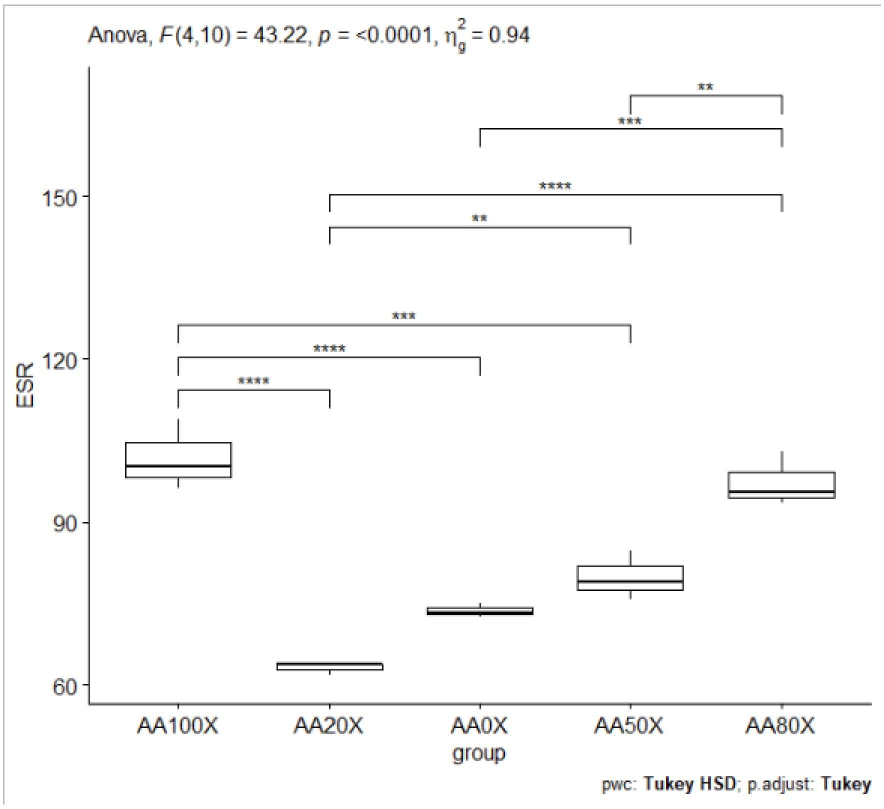

Figure S2. P.adjust dependence from Tukey test for significance.

ATR-IR of neat pIPNs samples

The ATR-FTIR spectra of the neat pIPNs between 600 and 2500cm^{-1}, which is the fingerprint region for these materials, are presented in Figure S3A. The band at ~1240 cm^{-1} in the spectrum of PAA can be assigned to -C-O- + OH in-plane bending of *syndiotactic* PAA. This band is shifted to 1248cm^{-1}, and to 1251cm-^{1} for samples AA100 and AA80 respectively[1]
, and completely disappears for the rest of the samples.

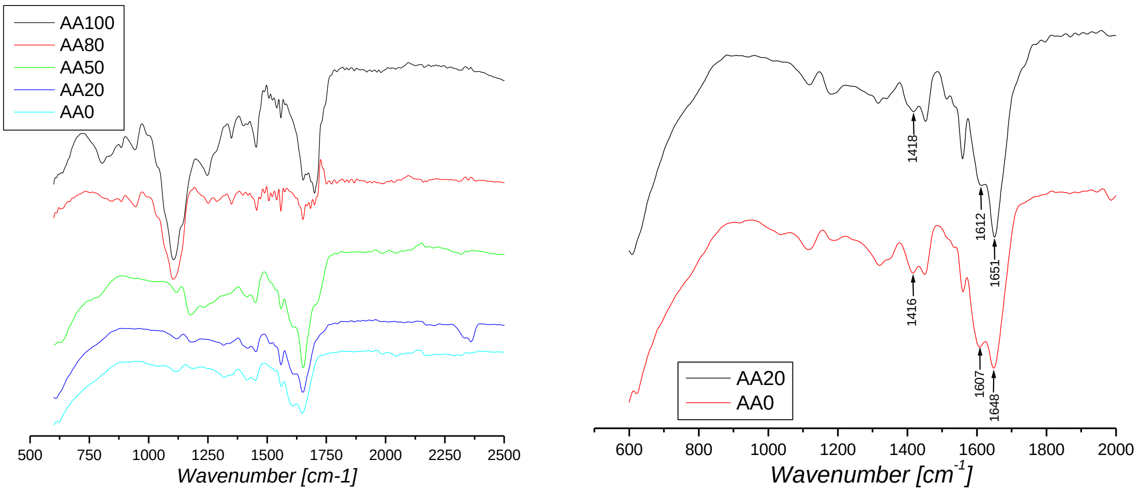

A **B**

Figure S3. ATR-IR spectrum of neat pIPNs (A) and samples AA0 and AA20, showing the band shifting due to hydrogen bonding (B).

In the spectrum of AA0, which is neat PAAM, the bands appearing at 1648 cm^{-1}, 1607 cm^{-1} and 1416 cm^{-1} can be assigned to $\nu_{C=O}$ Amide I, δ_{N-H} Amide II and δ_{C-N} Amide III, respectively.
In the spectrum of pIPN hydrogels the bands of Amide I and Amide III are shifted to 1653 cm^{-1} and 1418 cm^{-1}, respectively indicating the interaction of PAAM with PAA and the formation of hydrogen bonds between –COOH and –CONH$_2$ functional groups (Figure S3B). In addition, the band at 1607 cm^{-1} (δ_{N-H} Amide II) shifts to 1612 cm^{-1} (AA20 and AA50) and disappears in the spectra of samples AA80 and AA100.

Interaction of NaOH with the pIPNs components

The ATR-IR spectrums of the reference samples AA100N* and AA0N reveal that

(i) The Amide I band at 1648cm^{-1} shifts to 1658cm^{-1} i.e. the -C=O antisymmetrical stretching vibrations of PAA

(ii) Amide II band at 1608cm^{-1} completely disappears

(iii) Amide III band at 1416cm^{-1} shifts to 1404cm^{-1} i.e. -C=O symmetrical stretching vibrations of PAA.

These results show that PAAM undergo alkaline hydrolysis with formation of sodium polyacrylate (Figure S4);

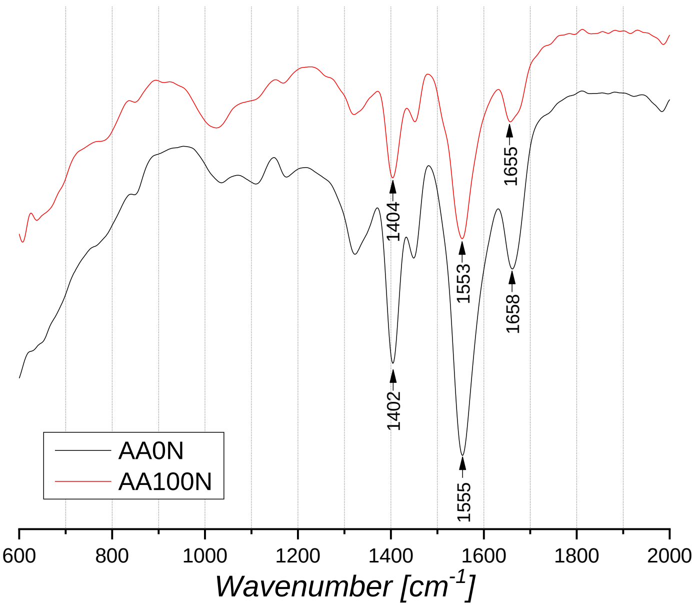

Figure S4. ATR-IR spectra of the pIPNs, obtained with PAA homopolymer (AA100N) and PAAM hopomopolymer (AA0N) after treatment with 6M NaOH for 72h

ATR-IR of reference samples AA100 and AA0

Interaction of Fe ions with pIPNs components

The ATR-IR spectra of the pIPNs, obtained with both homopolymers, namely homo-PAA (AA100N*) and homo-PAAM (AA0N) reveal that:

(i) The Amide I, II and III bands do not change their position when Fe ions enter the network. This is clearly seen when compare the spectra of AA0 sample with AA0Fe (Table S3, Figure S5A) and means that IR analysis cannot detect any interaction between PAAM and Fe ions, if any.

Table S3. Band assignments the pIPNS IR spectra with homopolymer PAAM (AA0 sample).

PAAM bands assignment[2]	Wavenumber [cm^{-1}]	AA0	AA0N	AA0Fe	AA0X
$\nu_{C=O}$ Amide I	1670	1648	1658	1649	1657
δ_{N-H} Amide II	1617	1608	disappear	1609	disappear
δ_{C-N} Amide III	1416	1416	1402	1416	1402

(ii) The bands of PAA at 1700 cm^{-1} and 1653 cm^{-1} for respectively -C=O antisym and $\nu_{C=O}$ –COOH shift to 1711cm^{-1} and 1666 cm^{-1} (Figure S5A, Table S4) which means that Fe ions preferably interact with –COOH groups of PAA than with –CONH$_2$ groups of AAM (Figure S5B, Table S4).

Table S4. Band assignments of the pIPNs with homopolymer PAA (AA100 sample).

PAA bands assignment [1, 3, 4]	Wavenumber [cm^{-1}]	AA100	AA100N	AA100Fe	AA100X
-C=O antisym.	1711	1700	1655	1711	1655
$\nu_{C=O}$ PAA	1650	1653	1655	1666	1655
-C=O sym.	1420	1417	1404	1417 int.↑	1404
-C-O- + OH in-plane bending Syndiotactic PAA	1240 1248 (Hydrogen bonded)	1248	disapp.	disapp.	disapp.
Isotactic PAA	930	943	disapp.	disapp.	disapp.
antisymmetrical vibrations of the ionized carboxyl group, COO^-Na^+	~1500	1558 very weak	1553	shifts to 1590 cm^{-1}	1549
antisymmetrical vibrations of the ionized carboxyl group, COO^-Fe^{2+}	1590	N.A.	n.a.	1584	disapp.
symmetrical vibration of the ionized carboxyl group, COO^-Fe^{2+}	1420	1417	n.a.	1417 int.↑	1404 int.↑

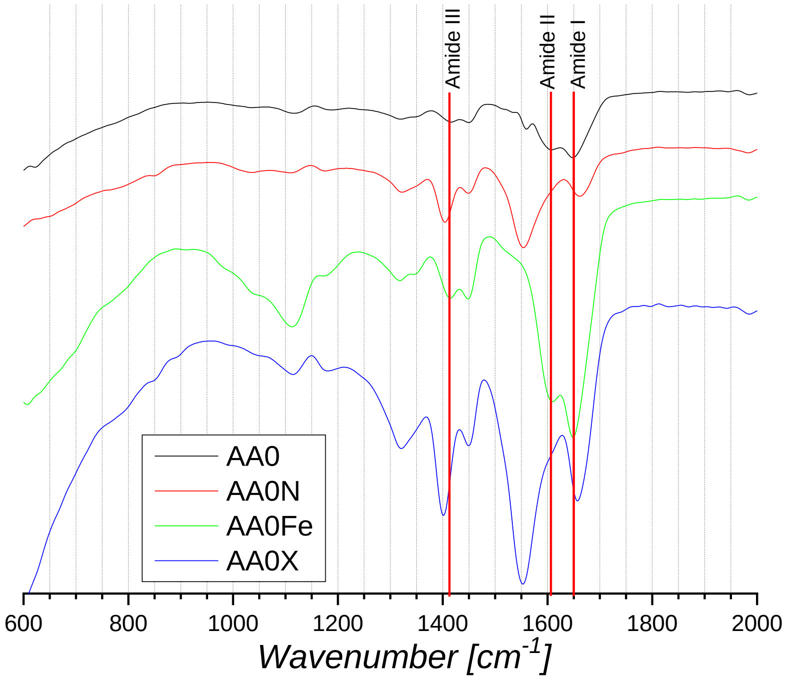

A

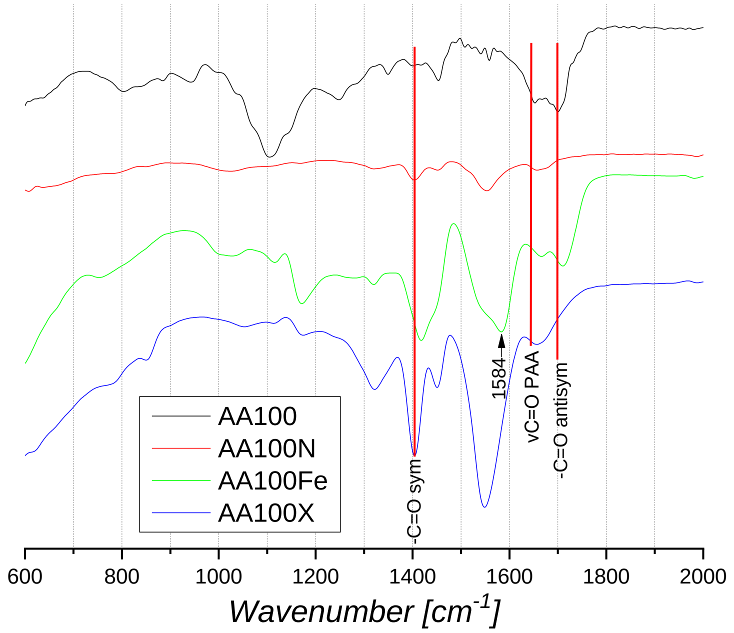

B

Figure S5. ATR-IR spectra of pIPNs (**A**) with homopolymer PAAM: neat (AA0); with absorbed Fe ions (AA0Fe); treated with 6M NaOH for 72 h (AA0N); and the respective magnetite/PAAM composite AA0X; and (**B**) with homopolymer PAA: neat (AA100); with absorbed Fe ions (AA100Fe); treated with 6M NaOH for 72 h (AA100N); and the respective magnetite/pIPNs composite (AA100X).

Equilibrium swelling ratio of pIPNs/magnetite composite hydrogels

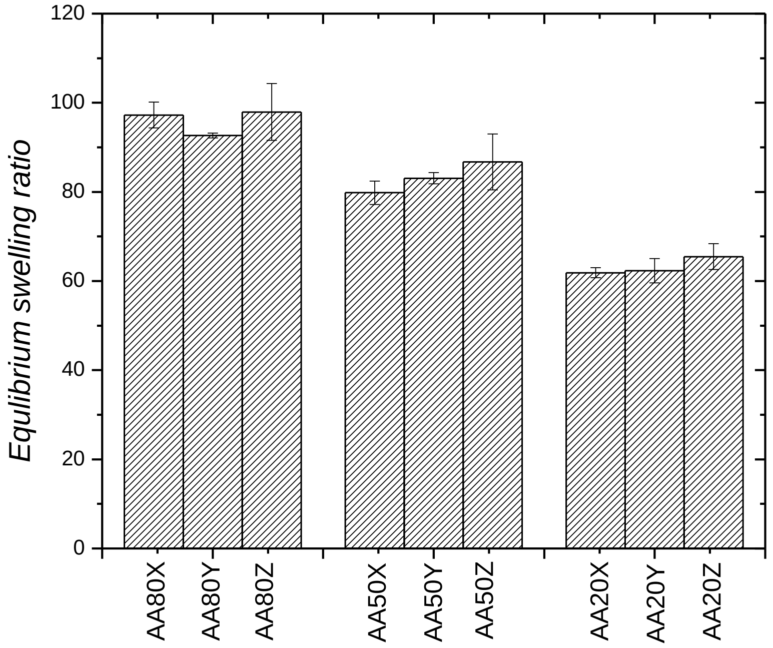

Figure S6. Equilibrium swelling ratio of pIPNs hydrogels with *in situ* formed iron oxide obtained via using different Fe aqueous solutions concentration: X (Fe^{3+} - 0.30 mol/l; Fe^{2+} - 0.15 mol/l); Y (Fe^{3+} - 0.030 mol/l; Fe^{2+} - 0.015 mol/l) Z (Fe^{3+} - 0.003 mol/l; Fe^{2+} - 0.0015 mol/l).

The data was further analyzed by Single factor ANOVA (Table S5)**.** The data analysis shows that the concentration of the the initial iron ions solution does not take influence on the ESR value of the resulting hybrid hydrogels.

Table S5. ANOVA analysis on the dependence of initial iron solution on the ESR of the resulting hybrid hydrogels.

Group 1	Group 2	*F*	*P-value*	*F crit*
AA80X	AA80Y	2,567195	0,184361	7,708647
AA80Y	AA80Z	0,651421	0,464861	7,708647
AA80X	AA80Z	0,000406	0,984884	7,708647
AA50X	AA50Y	1,370089	0,306774	7,708647
AA50Y	AA50Z	0,312319	0,606067	7,708647
AA50X	AA50Z	1,058256	0,361749	7,708647
AA20X	AA20Y	0,071823	0,801953	7,708647
AA20Y	AA20Z	0,625025	0,473419	7,708647
AA20X	AA20Z	0,651567	0,464814	7,708647

Iron content as determined by Flame Atomic Absorption Spectroscopy

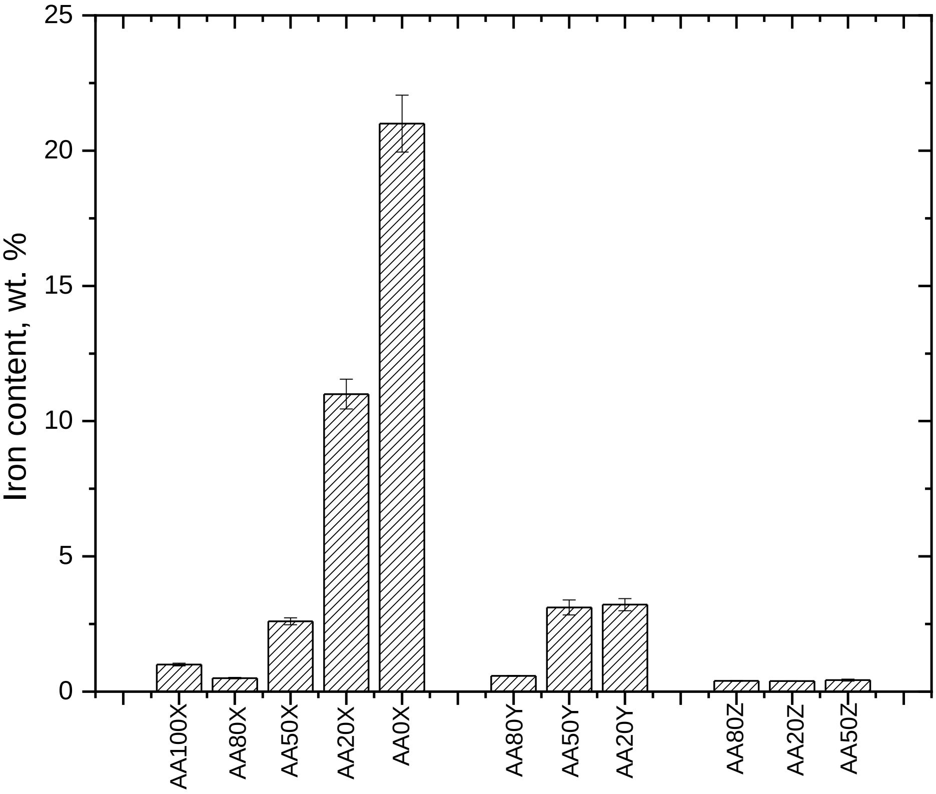

Figure S7. Iron content as determined by Flame Atomic Absorption Spectroscopy (FAAS) in pIPNs composite materials with *in situ* formed iron oxide obtained via using different Fe aqueous solutions concentration: X (Fe^{3+} - 0.30 mol/l; Fe^{2+} - 0.15 mol/l); Y (Fe^{3+} - 0.030 mol/l; Fe^{2+} - 0.015 mol/l) Z (Fe^{3+} - 0.003 mol/l; Fe^{2+} - 0.0015 mol/l).

X-ray diffraction of the pIPNs hybrid materials

Figure S8 presents the comparison between the XRD patterns of sample AA20X in its dry and swollen states. As it can be seen, in swollen state only the two most intensive peaks of magnetite are visible (denoted with asterisk) while the rest of the sample is completely amorphous since it contains nearly 99% water.

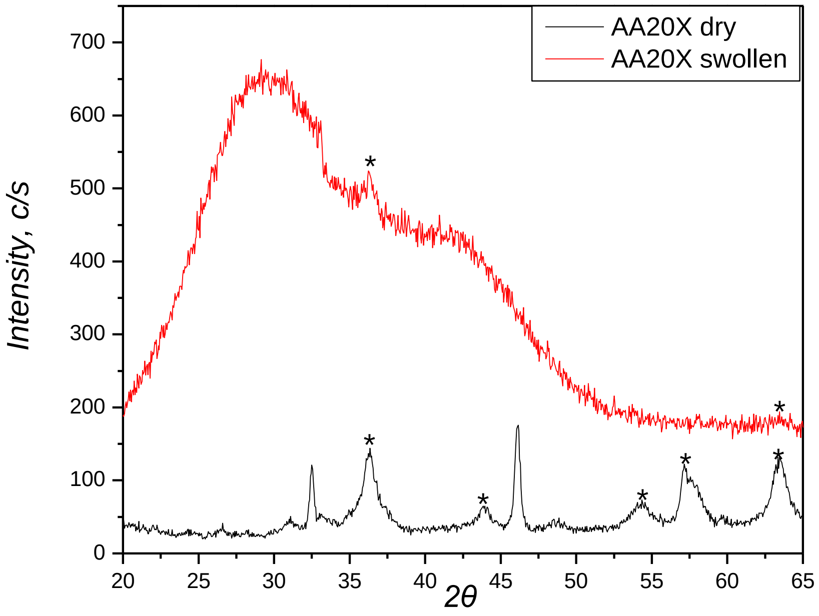

Figure S8. XRD patterns of sample AA20X in is dry and swollen state.

The influence of the Fe ions concentration in the initial solution used to *in situ* form the iron oxide in pIPNs is seen in Figure S8. The Fe concentration plays a key role for the *in situ* formation of magnetite: the lower the iron solution concentration is, the lower crystallinity of the obtained magnetite is observed. This is observed for the three studied pIPNs compositions, namely AA20, AA50 and AA80 (Figure S8). Thus, the interaction between the -COOH group from AA monomeric units and the Fe ions, which does not allow for the easy magnetite formation, is the reason for this observation.

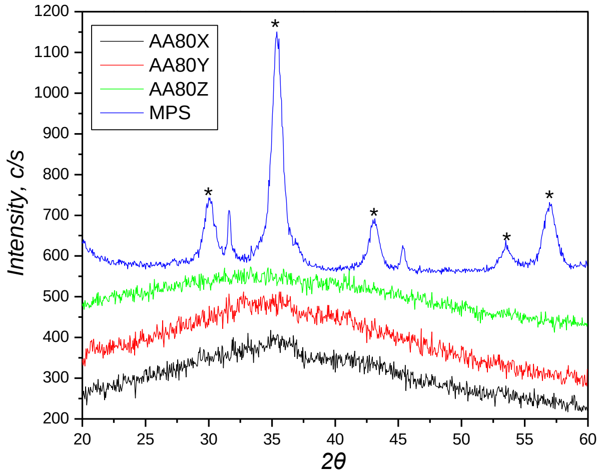

P(AA-co-AAM)/PAAM pIPN hydrogels AA80 with *in situ* formed magnetite

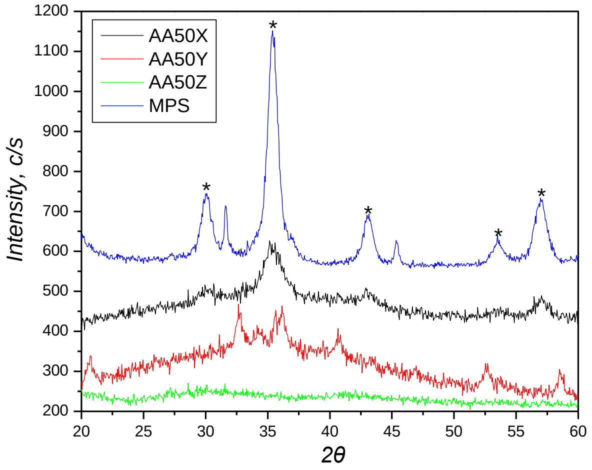

P(AA-co-AAM)/PAAM pIPN hydrogels AA50 with *in situ* formed magnetite

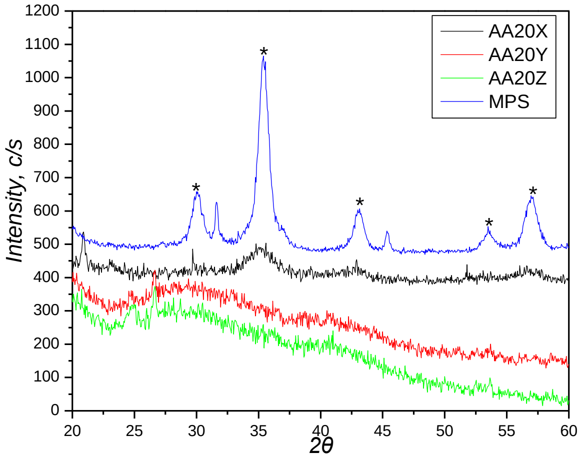

P(AA-co-AAM)/PAAM pIPN hydrogels AA20 with *in situ* formed magnetite

Figure S9. XRD diffractograms of pIPNs/magnetite composites with different composition with *in situ* formed magnetite.

Swelling ratios of pIPNs and the pIPNs/magnetite composites

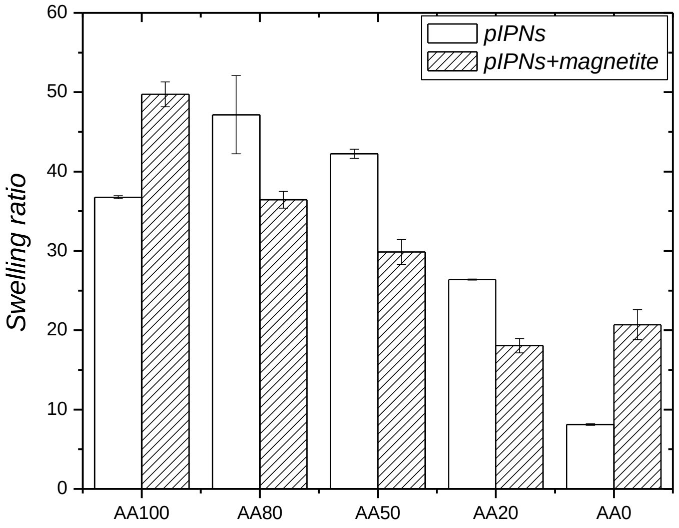

Figure S10. Comparison between the swelling ratios of pIPNs and the pIPNs/magnetite composites at pH=5.

Temperature responsive behavior of pIPNs/magnetite composites

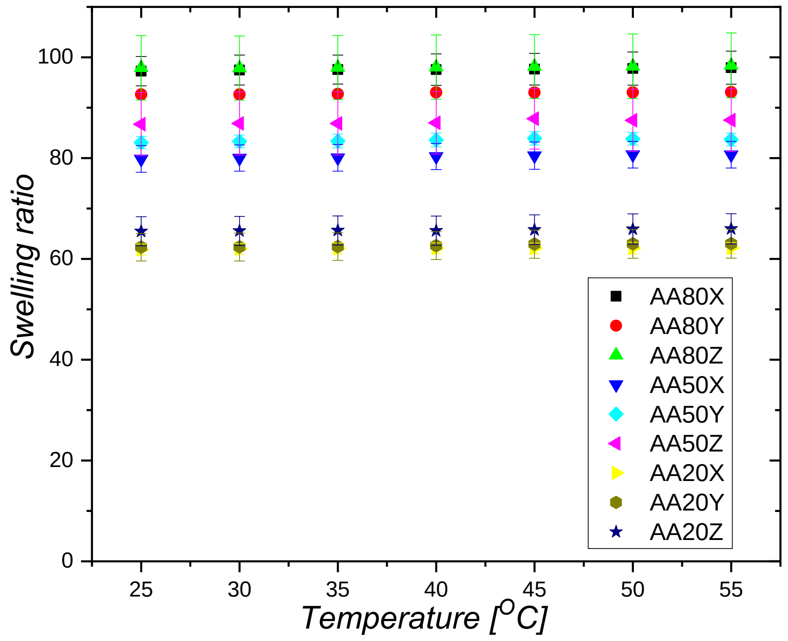

Figure S11. Temperature responsiveness of pIPNs/magnetite composite hydrogels.

Thermal properties

For neat PAAM/PAAM pIPN (AA0 sample), an endothermic peak appears at ~210 ºC which is due to the PAAM thermal decomposition.[5] The same peak appears also in the thermograms of pIPNs with low AA content, namely AA20 and AA50, however it is not observed for the pIPNs where PAA prevails (AA80 and AA100) (Figure S12). We have observed similar behaviour in our previous work where PAA/PAAM IPNs were studied.[6]

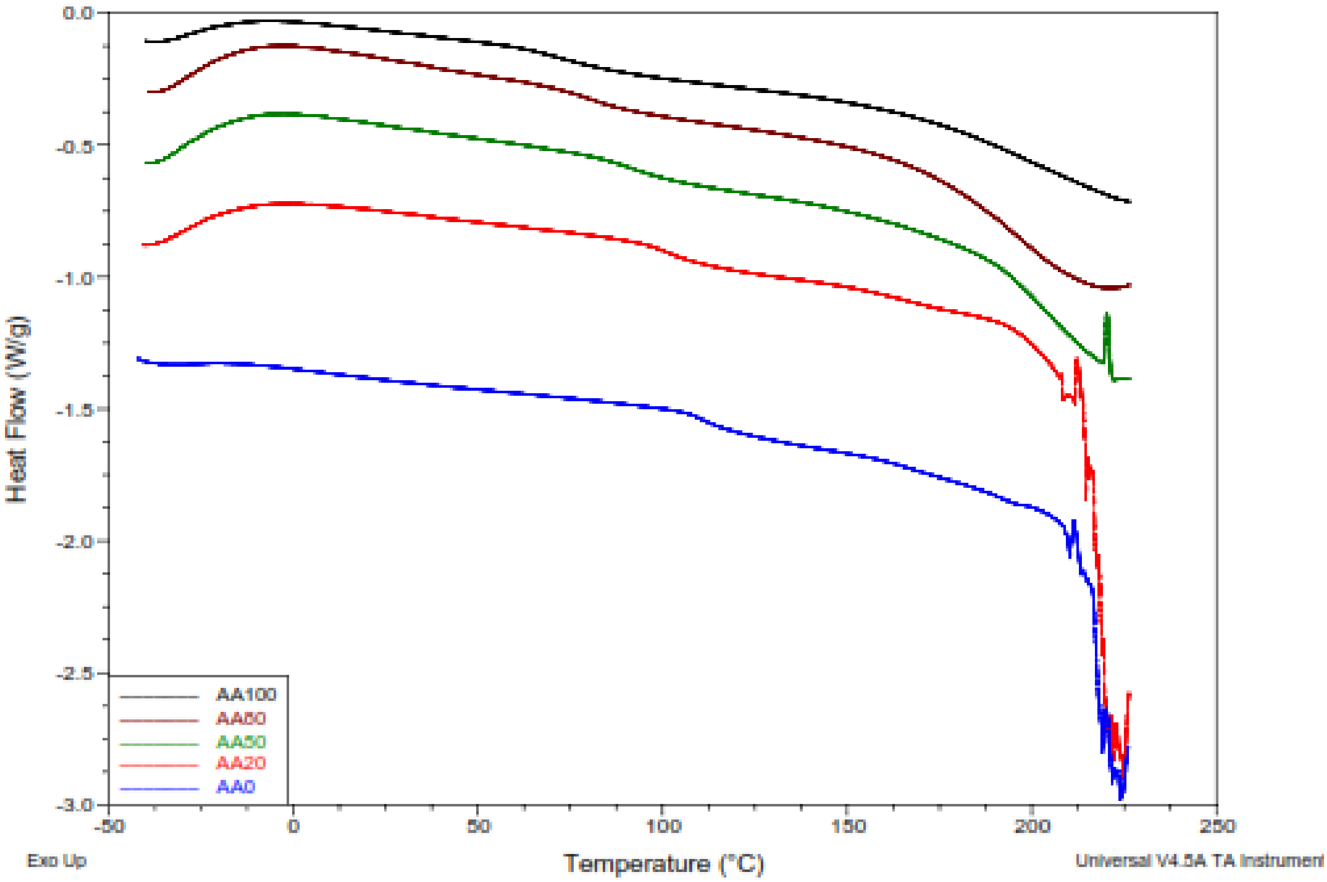

Figure S12. DSC thermograms of neat pIPNs (the 2nd heat scan from heat/cool/heat run is presented).

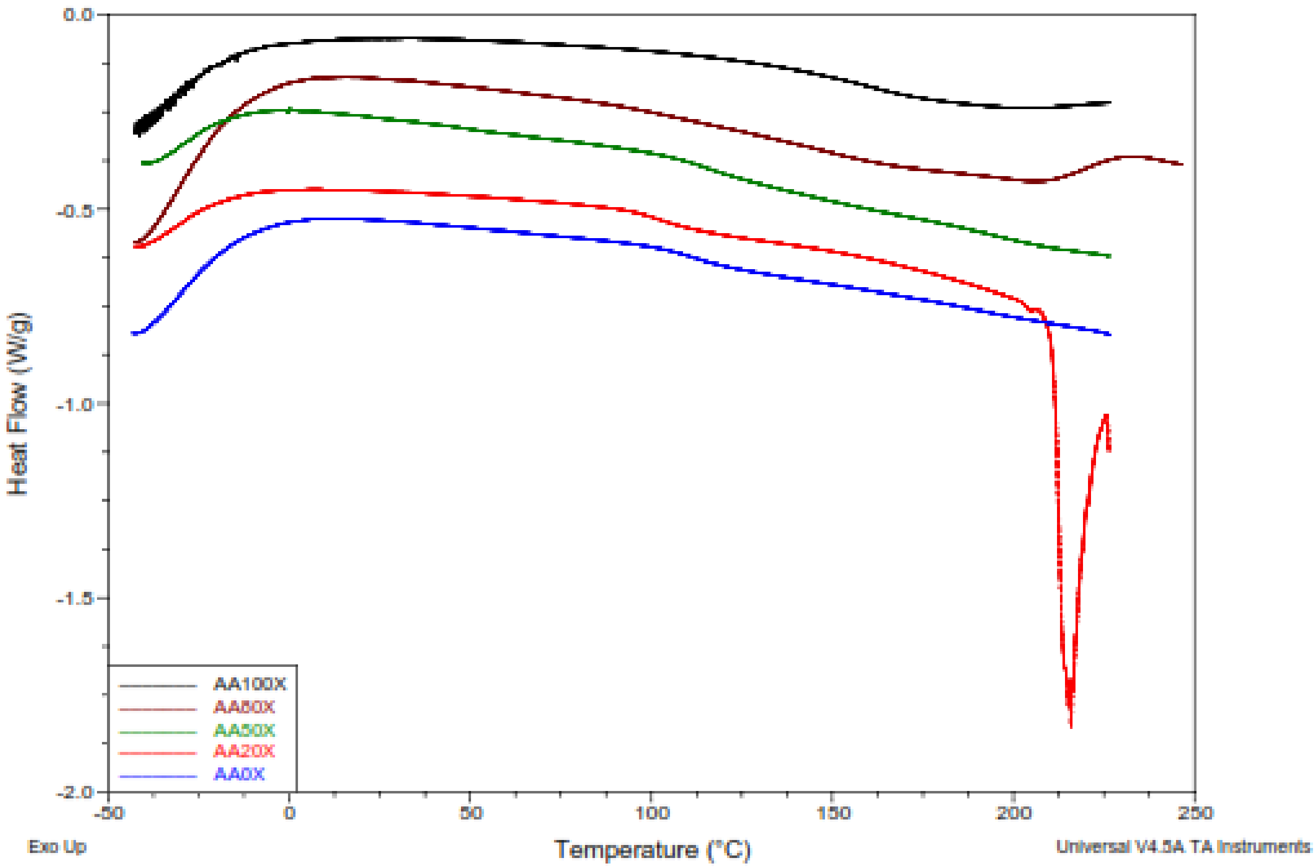

Figure S13. DSC thermograms of pIPNs/magnetite composites (the 2nd heat scan from the heat/cool/heat run is presented).

Table S6. Experimentally determined Tg of pIPNs and pIPNs/magnetite composites.

Sample	Tg [°C]
**AA100**	73
**AA100X**	161
**AA80**	80
**AA80X**	154
**AA50**	92
**AA50X**	115
**AA20**	102
**AA20X**	102
**AA0**	111
**AA0X**	111

Interaction of PAA with magnetite in the pIPNs/magnetite composites as revealed by ATR-FTIR

The composites were studied in their dry state. Figure S14 represents the "fingerprint" region of the spectrum. As they indicate there is no qualitative difference between them. It is shown PAAM undergoes alkaline hydrolysis to PAA with consequent neutralization to sodium polyacrylate (Figure S5.) as well as sodium polyacrylate does not interact with the *in situ* formed magnetite particles (Table S4, Figure S5) so the band shifting in the spectrum of neat pIPNs and the pIPNs/magnetite composites is related most probably to the transformation of the entire polymer matrix into sodium polyacrylate. This is in agreement by the work of other authors[12] and does not support the statements for electrostatic interactions between amide group and iron ions[7].

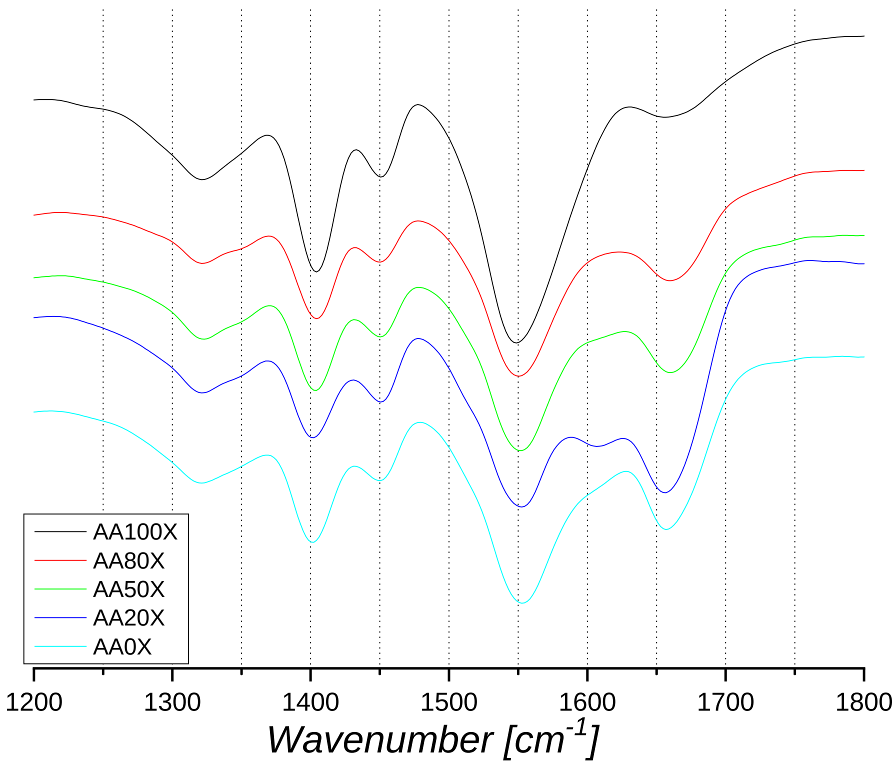

Figure S14. ATR-IR spectrum of pIPNs/magnetite composites. It is clearly visible there is no significant difference between samples.

Sorption and catalytic evaluation

The sample containing the highest amount of magnetite (AA0X) was tested as a potential adsorbent for tetracycline hydrochloride (TC). The experiments were conducted as follows: a certain amount of the material (approximately 10 mg dried sample) was immersed in 100 mL 10 ppl solution of tetracycline hydrochloride and the solution was bubbled with air (10 mL/min) to ensure homogenization. At certain times, a 2 mL aliquot sample was extracted from the solution and measured

on a UV-VIS spectrophotometer at 354 nm to determine the TC-HCl concentration then put back again in the reaction vessel.
The same procedure was applied for the determination of the persulfate activation performance with the difference of adding $K_2S_2O_8$ (concentration of 1mM in the reaction mixture).

The carried experiments show that the AA0X hybrid pIPNs/magnetite hydrogels do not appear to have adsorbtion properties toward TC. Previous work[7] reveals that magnetite particles solely can reduce the TC amount with ~30% in the course of the experiment while the persulfate/magnetite system reduce the TC amount nearly at full. The lack of such sorption properties can contribute to drug delivery systems based on hydrogels susceptible to changes in the magnetic field with no oxidative activity toward bioactive molecules thus providing the possibility for the development of smart delivery systems or biosensors.

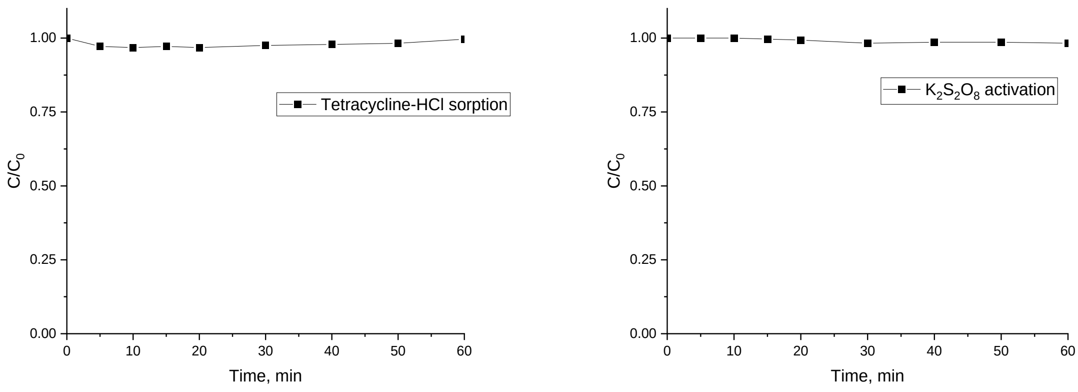

A **B**

Figure S15. Normalized concentration of TC removed by AA0X (**A**) and by of potassium persulfate activation (**B**)

DLS characterization

The size of the neat magnetite particles MPS (Table 2) was determined using Malvern 4700 system equipped with COHERENT innova70 laser working at λ=488 nm, at 90°. The measurements for each sample were triplicated and their averaged value is used.

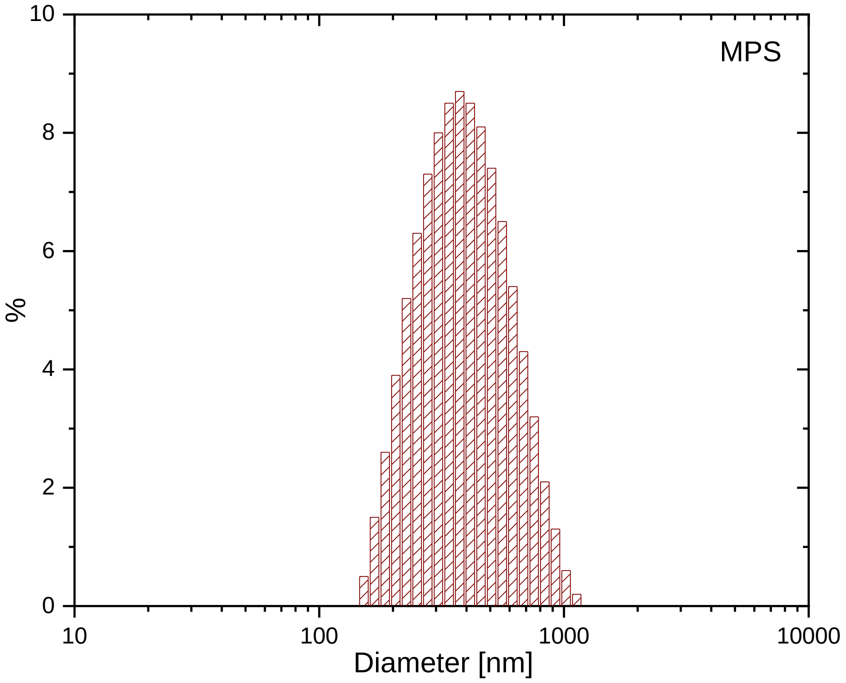

Figure S16. Size of the neat magnetite particles MPS as revealed by DLS.

Flame Atomic Absorbance Spectroscopy (FAAS)

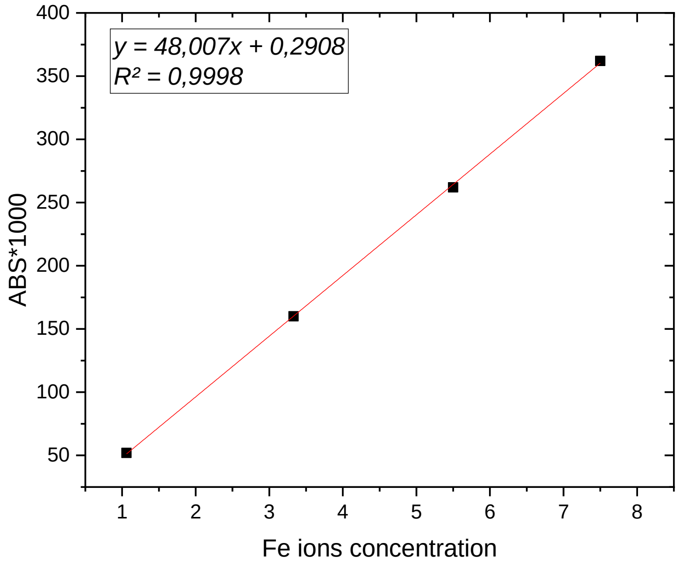

Figure S17. Calibration curve for Fe^{n+} ions, used in FAAS experiments.

---

[1] Lindén, L.-åke; Rabek, J. F. Structures and Mechanisms of Formation of Poly(Acrylic Acid)-Iron (II and III) Chloride Gels in Water and Hydrogen Peroxide. *Journal of Applied Polymer Science* **1993**, *50* (8), 1331–1341.

[2] Filho, E.; Brito, E.; Silva, R.; Streck, L.; Bohn, F.; Fonseca, J. Superparamagnetic Polyacrylamide/Magnetite Composite Gels. *Journal of Dispersion Science and Technology* **2020**, 1–9.

[3] Alvarez-Gayosso, C.; A. Canseco, M.; Estrada, R.; Palacios-Alquisira, J.; Hinojosa, J.; Castano, V. Preparation and Microstructure of Cobalt(Iii) Poly (Acrylate) Hybrid Materials. *International Journal of Basic and Applied Sciences* **2015**, *4* (3), 255.

[4] Namdeo, M.; Bajpai, S. K.; Kakkar, S. Preparation of a Magnetic-Field-Sensitive Hydrogel and Preliminary Study of Its Drug Release Behavior. *Journal of Biomaterials Science, Polymer Edition* **2009**, *20* (12), 1747–1761.

[5] Leung, W. M.; Axelson, D. E.; Van Dyke, J. D. Thermal Degradation of Polyacrylamide and Poly(Acrylamide-Co-Acrylate). *Journal of Polymer Science Part A: Polymer Chemistry* **1987**, *25* (7), 1825–1846.

[6] Simeonov, M. S.; Apostolov, A. A.; Vassileva, E. D. In Situ Calcium Phosphate Deposition in Hydrogels of Poly(Acrylic Acid)–Polyacrylamide Interpenetrating Polymer Networks. *RSC Advances* **2016**, *6* (20), 16274–16284.

[7] Lee, D.; Kim, S.; Tang, K.; De Volder, M.; Hwang, Y. Oxidative Degradation of Tetracycline by Magnetite and Persulfate: Performance, Water Matrix Effect, and Reaction Mechanism. *Nanomaterials* **2021**, *11* (9), 2292.
